# The equity implications of pecuniary externalities on an electric grid

**DOI:** 10.1093/pnasnexus/pgaf356

**Published:** 2025-12-09

**Authors:** Charles Sims, Gasser G Ali, J Scott Holladay, Tim Roberson, Chien-fei Chen, Islam H El-adaway

**Affiliations:** Department of Economics, Baker School of Public Policy, University of Tennessee, 1640 Cumberland Ave, Knoxville, TN 37996, USA; Department of Civil Engineering, University of Texas Rio Grande Valley, Edinburg, TX 78539, USA; Department of Economics, University of Tennessee, Knoxville, TN 37996, USA; Department of Economics, Finance, and Marketing, Tennessee Tech University, Cookeville, TN 38505, USA; Department of Sociology, Anthropology, Criminal Justice, Clemson University, Clemson, SC 29631, USA; Department of Civil, Architectural, and Environmental Engineering/Department of Engineering Management and Systems Engineering, Missouri University of Science and Technology, Rolla, MO 65409, USA

## Abstract

The adoption of rooftop photovoltaic (PV) systems can create upward pressure on retail electricity rates as utilities are forced to spread their fixed costs of generation and transmission across a smaller customer base. Since high-income households are more likely to purchase PV systems, low-income households may be disproportionately impacted by these rate increases. Using a novel combination of agent-based computational economic modeling and a choice experiment of rooftop solar adoption, we show how this pecuniary externality between low- and high-income customers increases low-income electricity bills by 10% in an area with some of the highest poverty rates in the United States. Since high-income solar adoption is less sensitive to electricity bills than low-income adoption, this pecuniary externality also reduces PV adoption inequity by nearly 1 percentage point. However, the reduction in PV adoption inequity, and the bill savings it generates, are not large enough to offset the $7.8 million ($9.86 per customer) annual increase in low-income electricity bills. Low-income assistance programs will likely fail to fully internalize the pecuniary externality due to horizontal and within-income-group vertical equity concerns.

Significance Statement On-site renewable energy generation is becoming an increasingly attractive investment; especially for high-income electric utility customers. How will low- and middle-income customers be affected by this trend? We study this question in the context of rooftop solar adoption in a region with some of the highest poverty rates in the United States. We show that high-income solar adoption increases the electricity bills of low- and middle-income customers in most cities in our study. Our findings are relevant for utility managers and policy makers concerned about the equity implications of the ongoing transformation of the electric grid.

## Introduction

The electric power industry is experiencing technology, policy, and economic changes that promise to drastically alter the way electricity is generated, transported, and consumed. Renewable energy is becoming a larger part of generation portfolios due to the need to reduce carbon emissions from the electricity sector and the lower costs of wind and solar technologies. At the same time, customers are investing in on-site generation which triggers a shift from the traditional hub-and-spoke model of electricity generation to a more distributed model where generation occurs closer to where the electricity is consumed. Previous research has investigated the environmental and efficiency implications of these shifts, but the distributional consequences are only now being recognized. High-income households are more likely to adopt rooftop photovoltaic (PV) systems than low- and middle-income (LMI) households (1–8) shifting the distribution of costs and benefits. The adoption of photovoltaics by high-income households forces utilities to raise residential rates to cover their fixed costs, which include any periodic cost that does not vary with electricity production, including debt service for past investments, operations and maintenance costs, and administrative expenses. Residential rates must rise in response to solar adoption when (i) PV adopters physically disconnect from the grid (grid defection) or (ii) utility fixed costs are recovered through volumetric retail rates instead of fixed grid access fees. If either of these conditions hold, high-income PV adoption will cause LMI households to face disproportionately higher energy bills (9–13).

These distributional concerns about solar adoption, often referred to as cross-subsidization or cost shifting, represent a pecuniary externality between high- and low-income customer groups. A pecuniary externality is when an agent’s actions cause a change in market prices which affect another agent (14–16). Unlike traditional externalities like air pollution, pecuniary externalities are not typically a cause for concern since they do not lead to efficiency losses (17). In fact, pecuniary externalities are a source of Joseph Schumpeter’s (18) famous “creative destruction.” However, when pecuniary externalities flow between high- and low-income groups, they can lead to “equity destruction” where LMI households shoulder a larger share of the costs of cleaning the electricity sector. We show how this pecuniary externality raises low-income electricity bills in an area with some of the highest poverty rates in the United States. We also demonstrate how a sufficient degree of sensitivity to the pecuniary externality can lower LMI electricity bills by encouraging more solar adoption.

To determine the full implications of pecuniary externalities, four questions must be answered. First, how much do electricity rates rise in response to distributed solar adoption? The distribution of customer preferences for solar across the physical network of transmission and generation assets (both centralized and decentralized generation) will determine where prices are likely to increase and by how much. Second, will these higher prices trigger additional solar adoption (a phenomenon known as a utility death spiral). While higher prices imply greater energy bill savings from solar systems, solar adoption is also influenced by the up-front cost of the system and forgone environmental impacts from burning fossil fuels. How customers weigh these adoption considerations will influence the magnitude of any utility death spiral. Third, is LMI solar adoption more sensitive to energy bill savings than high-income adoption? If so, the utility death spiral can increase solar adoption equity by incentivizing more LMI solar adoption. If not, the utility death spiral can result in a solar equity death spiral in which current solar inequity creates future solar inequity. Fourth, do the cost savings experienced by LMI solar adopters outweigh the additional costs imposed on LMI nonadopters from higher electricity rates? If so, higher retail electricity rates may actually reduce LMI electricity bills.

**Fig. 1.**
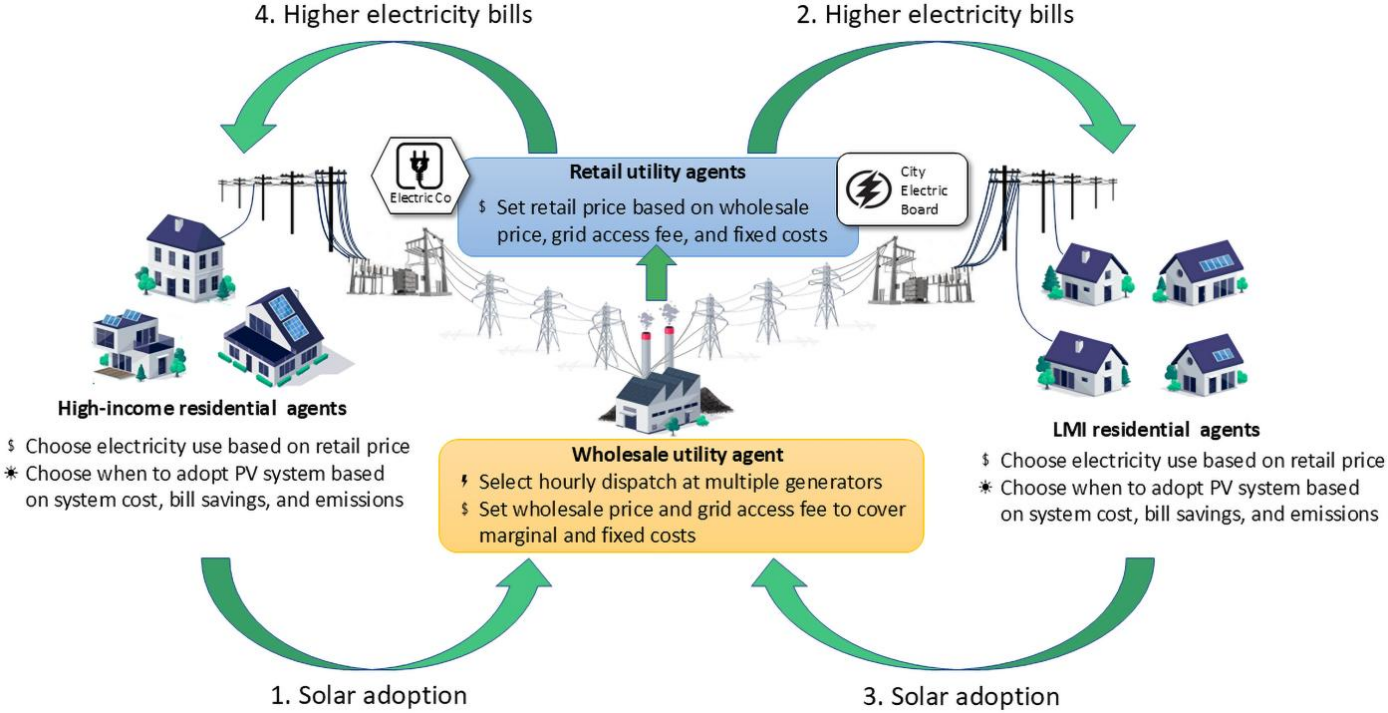
Our bottom-up agent-based approach unpacks aggregate supply and demand curves by focusing on the behavior of individual agents that mange grid components, set prices, consume electricity, and adopt solar. A utility death spiral amplifies PV adoption by high-income (linkages 1 and 4) and LMI (linkages 3 and 2) customers through adjustments in electricity rates. A pecuniary externality created by high-income solar adoption redirects the utility death spiral effect onto LMI customer (linkages 1 and 2). A similar pecuniary externality is created by LMI solar adoption (linkages 3 and 4). Image credit: iStock.

To address these questions, we use a novel combination of agent-based computational economic (ACE) modeling and a choice experiment of rooftop solar adoption to simulate the implications of a pecuniary externality between high- and low-income electricity customers. The ACE model is a dynamic model of the electricity market in which heterogeneous customer and utility agents interact by impacting and adapting to market clearing prices generated through an electricity transmission model that accounts for grid congestion (19). Customer agents buy electricity from retail utility agents who buy electricity from a single regulated wholesale utility agent who chooses generation at power plants to minimize the cost of supplying electricity (Fig. 1).

**Fig. 2.**
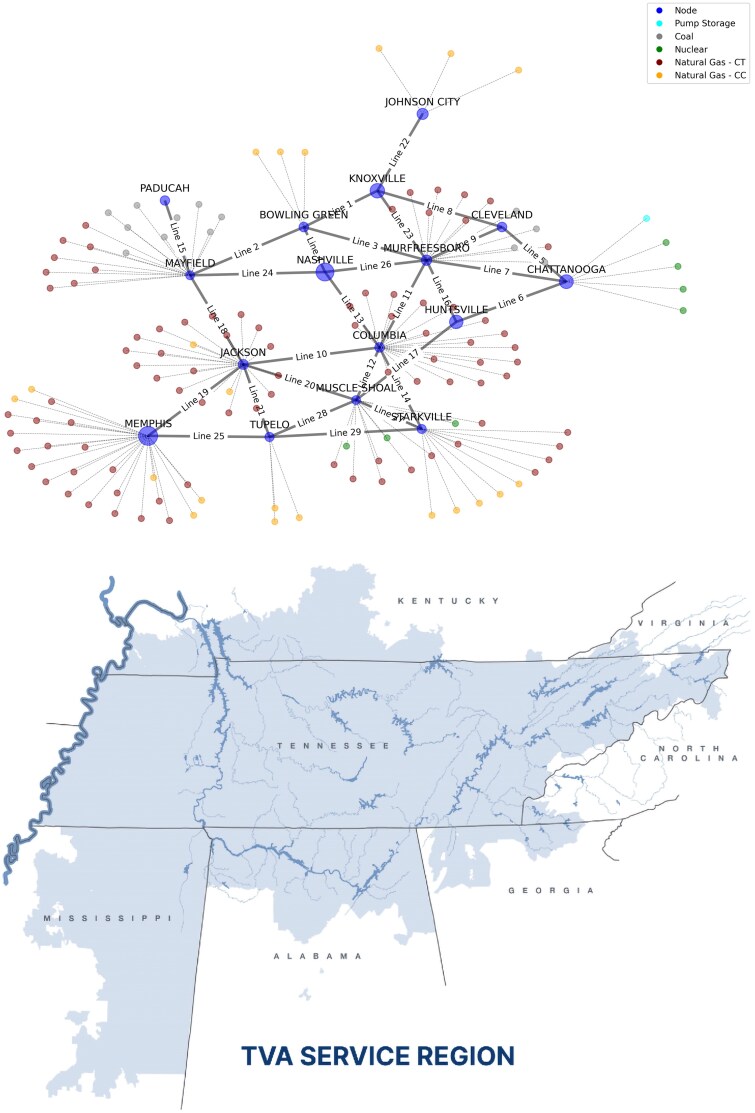
Representation of the TVA grid within our ACE model of a regulated power market. Network nodes correspond to TVA’s 16 Customer Service Districts with larger circles indicating larger customer bases. Each dot indicates the location of a TVA generating unit (coal, nuclear, natural gas) or pump storage facility.

We apply our model to the Tennessee Valley Authority (TVA) (Fig. 2); a region with some of the highest poverty rates in the United States. Like many areas across the United States, rooftop solar adoption rates in the region are currently low limiting the insight gained from traditional empirical methods. However, TVA is an ideal application of our ACE model because TVA’s residential customers are very heterogeneous along several socioeconomic dimensions ranging from historically poor parts of Appalachia in the east to rural, agricultural communities in the west with large cities dispersed throughout. This socioeconomic mix makes for an ideal testbed for studies on the equitable distribution of distributed solar adoption impacts.

We then adapt high-income and LMI residential customer agent behavior in the ACE model by introducing region-specific PV adoption rules fit to the results of a choice experiment of 2,307 TVA residential customers. The results of the choice experiment identify how the willingness to pay for rooftop solar varies with three characteristics of the PV system: (i) upfront cost, (ii) savings on electric bills, and (iii) reductions in emissions that occur from reduced reliance on fossil fuel generation. To generate a positive relationship between PV adoption and retail rates in the ACE model, we focus on a case where customers only consider battery-plus-solar systems, allowing them to defect from the grid following adoption.

The result is a tightly coupled model in which the equilibrium electricity prices and carbon emissions generated by the ACE model triggers regional variation in solar adoption that subsequently alters electricity prices and carbon emissions. This bottom up approach allows us to capture distributional impacts of solar adoption by accounting for heterogeneity in demand for electricity and solar, regional variation in retail electricity prices and carbon emissions from electricity generation, and interactions between customer agents mediated by markets and the physical transmission network. While agent-based models have been applied to study energy transitions generally (20–24) and solar adoption specifically (25, 26), we are the first to leverage the benefits of integrating agent-based models and choice experiments to study the equity implications of grid decarbonization (27).

## Solar adoption and pecuniary externalities

Solar adoption is subject to a behavioral feedback loop known as a “utility death spiral” that arises from the interaction of utility and customer agents. Solar adoption lowers demand during daylight hours which shifts relative demand across hours of the day and regions on the grid. In response, a utility agent adjusts dispatch at each generator, which triggers adjustments to wholesale and retail prices, and the carbon intensity of grid power, which triggers subsequent adjustments to the customers’ solar adoption rates. Utilities bear sunk investment costs in generation and transmission assets, in addition to operations and maintenance costs that are not directly tied to the marginal cost of electricity generation (11). If these nonmarginal costs of building and operating an electric grid are large, customer solar adoption can lead to higher retail rates as those fixed costs must be spread over a smaller group of customers.

The strength of this feedback loop varies because each customer agent weighs cost, bill savings, and environmental considerations differently. For example, low-income residential agents may weigh the upfront cost more than high-income customers motivated by reducing their carbon footprint. Likewise, a customer paying relatively high retail prices will be more influenced by bill savings than a customer paying low retail prices. Figure 1 shows the utility death spiral effect for high-income (linkages 1 and 4) and LMI (linkages 3 and 2) customers.

A pecuniary externality (cost shifting) redirects the utility death spiral effect onto another customer group. A pecuniary externality from high-income to LMI customers is depicted as linkages 1 and 2 in Fig. 1 while a pecuniary externality from LMI to high-income customers is depicted as linkages 3 and 4. With PV adoption inequity, the pecuniary externality raises bills on an increasingly LMI customer base. If solar adoption is sensitive to electricity bill savings, the pecuniary externality will incentivize more LMI solar adoption (linkages 1–3 in Fig. 1) and lower overall LMI electricity bills.

## Application to the Tennessee Valley Authority

Our stylized TVA grid is depicted in Fig. 2. Retail utilities correspond to TVA’s 16 Customer Service Districts represented by the blue dots. Each retail utility is composed of four customer agent types (industrial, commercial, LMI residential, and high-income residential) for a total of 16×4=64 customer agents each with a distinct demand profile and likelihood of solar adoption. Customers only consider battery-plus-solar systems so that adoption manifests as a reduction in customers rather than a change in the hourly load. An attractive feature of this modeling choice is that PV system size (and cost) is determined endogenously by residential electricity demand and will vary across LMI and high-income agents.

While TVA’s grid includes thousands of buses and transmission lines, limited customer data necessitates that we consolidate buses and transmission lines into a more compact grid. One grid node or bus is located in each retail utility, with 29 transmission lines connecting each retail utility each with a different capacity and reactance. One hundred and forty-one coal, nuclear, natural gas, and hydro generating units are spread across the 16 network buses. The size, fuel type, and geographic distribution of the generation supply is represented by the number and color of dots connected to each bus by the thin gray lines.

## Agent-based simulation of solar adoption

In this section we use different model simulations to isolate the implications of a pecuniary externality between high income (those earning $150,000 or more annually) and LMI customers (<$150,000 annually). We first develop a 30-year benchmark simulation (2021–2051) with retail prices that periodically adjust, a solar rebate that reduces PV system cost by 26% (roughly equal to the federal rebate program prior to 2021), solar irradiance typical of much of the Tennessee Valley (4 sun hours), and TVA’s scheduled coal-fired power plant retirements. The model predicts ∼0.2% of TVA customers adopt solar which is consistent with the low amount of solar adoption in the TVA service area currently. As the cost of solar systems falls and electricity rates rise, adoption in our benchmark simulation steadily increases over time (Appendix  Figure 6). By 2051, TVA’s customer base drops by 30%.

Table 1 shows that the 30-year adoption rate varies from a low of 12% in Mayfield, KY to a high of 40% in Tupelo, MS. These differences in adoption are driven by two factors in our model. First, the regional differences in retail electricity prices that dictate the potential cost savings from adopting rooftop solar. In 2021, retail prices vary from less than $0.08/kWh in Huntsville, AL to over $0.10/kWh in Memphis, TN. Second, results from the choice experiment indicate that customers in some regions value solar panels more than others and are willing to pay more.

**Table 1.** Adoption rate (% solar adoption) between 2021 and 2051 and the resulting adoption gap between high income and LMI customers (in percentage points)

	All residential (%)	High income (%)	LMI (%)	Adoption gap (pp)
TVA service area	30	35	30	5.4
Tupelo, MS	40	79	38	41.7
Starkville, MS	36	34	36	−1.6
Paducah, KY	32	29	33	−3.4
Nashville, TN	28	31	27	3.8
Muscle Shoals, AL	12	65	9	55.8
Murfreesboro, TN	23	18	24	−5.4
Memphis, TN	37	43	36	6.8
Mayfield, KY	12	0	12	−12.3
Knoxville, TN	21	21	21	0.2
Johnson City, TN	24	78	20	58.2
Jackson, TN	33	19	34	−14.9
Huntsville, AL	36	32	37	−5.1
Columbia, TN	22	44	21	23.4
Cleveland, TN	29	79	25	53.2
Chattanooga, TN	29	34	28	5.9
Bowling Green, KY	37	40	37	2.9

## Measuring the utility death spiral

A portion of the adoption in Table 1 is driven by the utility death spiral. The declining load due to solar adoption triggers two countervailing effects. First, the grid becomes less congested thereby lowering the marginal cost of delivering electricity to the retail utilities. Second, the fixed costs must be spread over fewer customers. From 2021 to 2051, average residential electricity bills in our benchmark simulation increased 10% across the TVA service area due to the increase in PV adoption with a range of 5% in Mayfield, KY to 20% in Huntsville, AL. The rise in retail prices suggest that the increases from fixed costs dominate the declining marginal costs.

To calculate how much solar adoption can be attributed to the utility death spiral, we run a counterfactual simulation where the wholesale and retail utility agents are unable to recoup their fixed costs through rate increases and instead charge a constant wholesale and retail price over our 30 year simulation. Such a scenario may arise when fixed costs are recovered via taxes, municipal bonds, or other government subsidies (28). With fixed retail prices, the rate of adoption between 2021 and 2051 is slightly lower (29.32%) since customers are sensitive to energy bills when making solar adoption decisions (Appendix  Figure 7).

Comparing the dynamic and fixed retail price simulations reveals that the death spiral is responsible for 11,155 additional TVA customers adopting solar over our 30 year simulation; a <1 percentage point increase in solar adoption. In other words, every customer that adopts solar generates 0.03 future adoptions due to the resulting increase in retail prices. The amount of adoption attributable to the utility death spiral varies across retail utilities but is always less than 1.5 percentage points (Appendix  Table 12).

## Equity implications of the pecuniary externality

Table 1 decomposes the benchmark simulation results into high-income and LMI customer groups. Nearly 35% of high-income customers adopt while only 30% of LMI customers adopt. This 5.4 percentage point adoption gap combined with the utility death spiral means high income customers are negatively impacting LMI customers through increases in electricity bills; both within and across retail utilities. This effect, captured by linkages 1–2 in Fig. 1, is consistent with cost shifting (9–13).

However, these increases in electricity bills spur additional solar adoption. The increase in rates due to the utility death spiral creates an additional 1,270 high-income solar adopters. With a death spiral multiplier of 1.03, these additional high-income adoptions generate 1,305 additional LMI adoptions via the pecuniary externality.

Equity concerns about rising electricity bills may be alleviated somewhat if this increase in LMI adoption lowers LMI energy costs through solar adoption. If the pecuniary externality is countering the economic, cultural, and structural hurdles to solar adoption among LMI households (6, 10, 29–33), then rising retail rates are a market mechanism for reducing solar inequity and LMI energy costs via an extensive margin adjustment. However, if the pecuniary externality is exacerbating adoption gaps, then the utility death spiral effectively creates a solar equity death spiral in which the adoption gap between high-income and LMI customers grows.

**Fig. 3.**
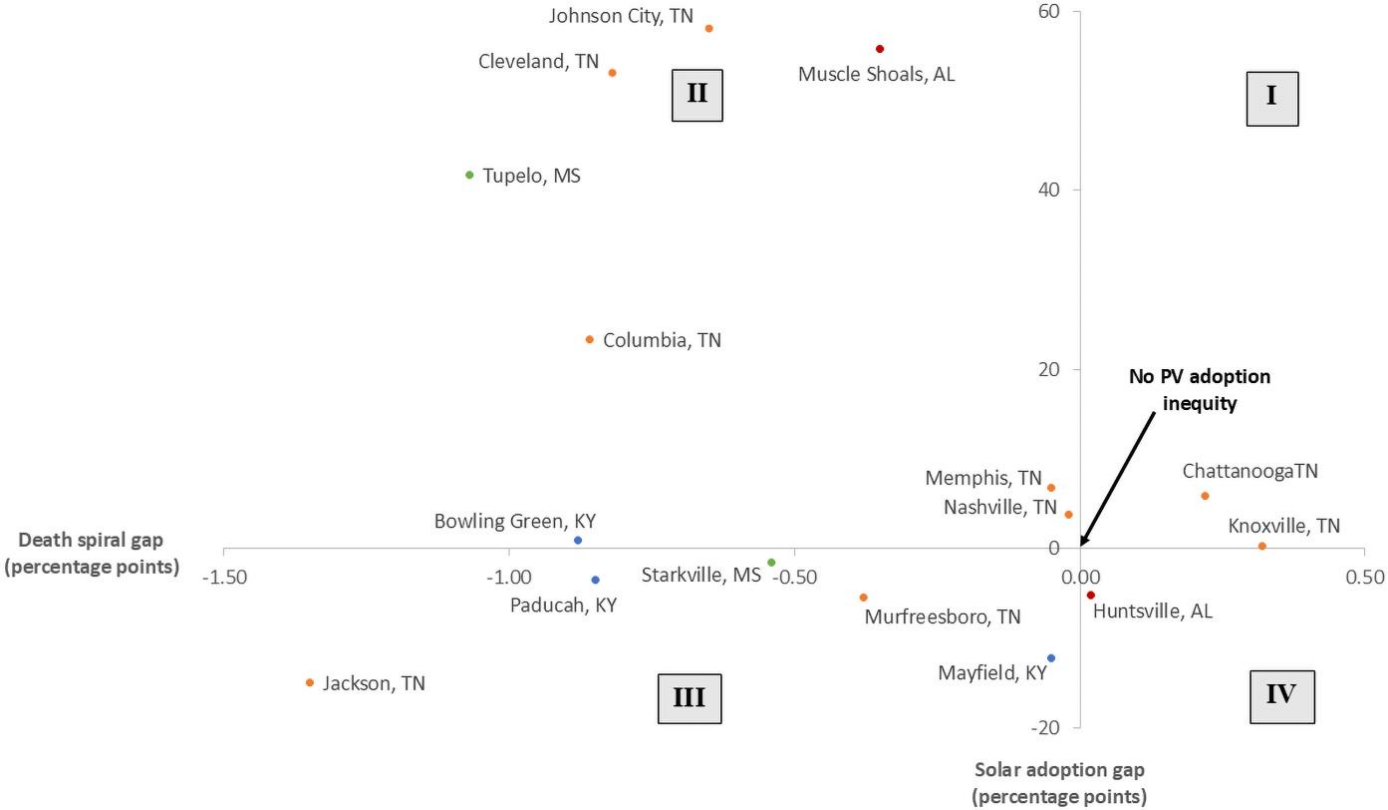
Equity implications of a pecuniary externality between high-income and LMI customers in the TVA service area. Solar adoption gap (y-axis) is the difference in the high-income and LMI solar adoption rate (% customer adoption) over our 30 years simulation. Death spiral gap (x-axis) measures the difference high-income and LMI adoption rate sensitivity to rising electricity prices.

To answer this question, we calculate the difference in the adoption rate in our benchmark simulation and a hypothetical fixed-rate simulation. We then compare adoption rates in these two cases for high-income and LMI customers. This death spiral gap is slightly positive indicating that rising retail electric rates triggered by the predominately high-income solar adoption is actually shrinking the solar adoption gap in the TVA service area. Once again, system-wide averages can obscure important heterogeneities across the grid. Figure 3 demonstrates the equity implications of a pecuniary externality between high-income and LMI residential customers. The vertical axis shows the difference in high-income and LMI solar adoption rate. A positive value indicates a higher rate of adoption by high-income customers while 0 indicates no adoption inequity. Quadrants I and II are cases where higher retail rates are disproportionately experienced by LMI individuals because of solar adoption gaps.

The horizontal axis measures the death spiral gap where positive values indicate that the increase in electricity prices has a bigger impact on high-income adoption than LMI adoption. In quadrant II, the pecuniary externality reduces solar inequity because LMI adoption is more sensitive to retail price increase than high-income adoption in these retail utilities. In other words, the adoption gap would have been higher without the 7–11% increase in retail electricity rates. In contrast, for the retail utilities in quadrant I (Chattanooga and Knoxville), the utility death spiral translates into a solar equity death spiral in which rising retail prices increase the gap between high-income and LMI adoption. In these retail utilities, high-income customers are more likely to adopt and this adoption creates a pecuniary externality that (i) raises electric bills for an increasingly LMI customer base and (ii) exacerbates the existing adoption gap. Similar dynamics occur in quadrants III and IV though PV adoption inequity has been less of a concern when high-income adoption lags LMI adoption. For example, the pecuniary externality in Huntsville shrinks the adoption gap by raising high-income adoption more than LMI adoption.

The decrease in the adoption gap comes at the cost of higher energy bills paid by LMI customers. Average retail rates in the TVA service area increased from 9.02 to 9.93 cents per kWh. To help utilities and policymakers evaluate the tradeoffs between higher energy bills and increased LMI solar adoption, we calculate the net change in LMI electricity bills due to the pecuniary externality (Table 2). LMI nonadopters across the TVA service area will pay an additional $7.8 million ($9.86 per customer) in energy bills in 2051 due to the pecuniary externality. However, the desire to avoid this increase in energy bills triggered 9,827 additional LMI solar adopters for a total savings of $976,073. On net, the pecuniary externality raises electricity bills for the LMI customer agent in each retail utility. However, if the utility death spiral spurred a 6.3 percentage point increase in the LMI adoption rate rather than the <1 percentage point increase from our benchmark simulation (see last column in Table 2), the pecuniary externality would lower electricity bills for LMI customers via an extensive margin adjustment—solar adoption.

**Table 2.** Effect of pecuniary externality on LMI electricity bills and the price responsiveness of LMI solar adoption needed for the pecuniary externality to lower LMI electricity bills (in percentage points)

	Increase in bills	Avoided bills due to solar adoption	Net LMI bill savings	Increase in LMI adoption rate needed to lower LMI bills (pp)
TVA service area	$7,774,460	$976,073	−$6,798,387	6.3
Tupelo, MS	$54,903	$9,970	−$44,932	6.1
Starkville, MS	$56,493	$6,575	−$49,918	7.7
Paducah, KY	$59,489	$13,702	−$45,788	7.5
Nashville, TN	$1,564,357	$171,067	−$1,393,290	6.1
Muscle Shoals, AL	$33,414	$1,805	−$31,608	10.0
Murfreesboro, TN	$285,931	$27,627	−$258,304	7.1
Memphis, TN	$1,868,109	$257,549	−$1,610,560	6.5
Mayfield, KY	$18,346	$244	−$18,102	7.2
Knoxville, TN	$1,089,636	$102,100	−$987,535	3.8
Johnson City, TN	$361,542	$36,118	−$325,424	5.0
Jackson, TN	$210,487	$37,148	−$173,338	7.9
Huntsville, AL	$1,135,606	$139,653	−$995,954	6.5
Columbia, TN	$102,116	$16,124	−$85,992	6.5
Cleveland, TN	$163,511	$18,365	−$145,146	4.4
Chattanooga, TN	$926,754	$131,932	−$794,822	6.0
Bowling Green, KY	$137,043	$19,444	−$117,599	6.4

To place this result in context, we consider two counterfactual simulations (see Appendix  Figures 8 and 9 and Table 13) that reveal that the 26% federal solar rebate is responsible for a 6 percentage point increase in residential adoption while an increase in solar irradiance comparable to southern California is responsible for an 8 percentage point increase. Thus, if LMI customers were as responsive to a 10% electricity price increase as they are to a 26% solar rebate, the pecuniary externality would lower LMI electricity bills. While recouping utility fixed costs via prices has been discouraged for equity reasons (9, 11, 34), we demonstrate how that design can reduce PV adoption inequity and provide a net reduction in LMI electricity bills if LMI customers are sufficiently sensitive to electricity bill savings.

## Discussion and conclusion

We find that a utility death spiral can create a pecuniary externality between high-income and LMI electricity customers. While pecuniary externalities are not typically a justification for market intervention, this one causes equity concerns since high-income solar adoption raises electricity rates on a customer base that is increasingly LMI. However, this pecuniary externality increases PV adoption equity across most of TVA’s electric grid. The exception is in two of TVA’s largest load centers, Knoxville and Chattanooga, where the utility death spiral may create a solar equity death spiral.

How should utilities and policy makers concerned about the equity implications of a transitioning electric grid respond to the pecuniary externality? First, it is important to note that the pecuniary externality flows from adopters to nonadopters. Like LMI customers, the increase in the energy bills of high-income nonadopters also exceeds the energy bill savings of solar installation for high-income adopters. Over 86% of solar adopters meet our definition of LMI in the TVA service area. Since most of these LMI adopters were likely at the upper end of the LMI income category, the externality is running from middle income customers to low- and high-income customers. These horizontal and within-income-group vertical equity concerns suggest that the winners and losers from policies designed to address cost shifting may not be easily demarcated by income. Low-income assistance programs may be justified based on other equity concerns such as energy burden but will be a second-best policy prescription to address cost shifting. However, winners and losers will need to be identified, as equity is the primary motivation to internalize the pecuniary externality. More responses from lower income customers to our choice experiment would have permitted our analysis to consider low, middle, and high-income customer groups to provide a more nuanced picture of the equity concerns raised by the pecuniary externality.

It is also important to remember that the pecuniary externality will be smaller if customers do not defect from the grid. Eliminating grid defection will allow the utilities to recover their fixed costs from the entire customer base which will dampen the per-unit retail price increase. With no grid defection, charging end-use customers a grid access fee in addition to a per-unit price (a two-part tariff) could eliminate the pecuniary externality entirely. However, in practice, utilities may be reluctant to charge customers a grid access fee large enough to recoup all the utility’s fixed costs and may continue to recoup part of their fixed costs through the per-unit price.

In settings where grid defection is common or there is resistance to two-part tariffs, our results highlight other policy options. To correct externalities, policies should be designed to internalize external costs. If equity is a policy objective, the pecuniary externality should be addressed the same way. PV adopters should be forced to internalize the higher electricity costs they force onto nonadopters when choosing to adopt rooftop PV. Charging PV adopters a higher per-unit cost of electricity after they adopt or reducing the feed-in tariff that PV adopters receive from utilities are both consistent with internalizing the external costs of adoption. In practice, the marginal external cost of PV adoption will vary over space and time and will be difficult to estimate. Nevertheless, the pecuniary externality combined with PV adoption incentives will lead to inequitable outcomes, particularly since solar has been shown to be over-subsidized relative to its social benefits (e.g. (35)).

While our model is based on the TVA service area, the heterogeneity in solar adoption rates and the adoption gap between high-income and LMI customers is likely similar in other areas with low PV penetration. Heterogeneous adoption of PV will lead to a similar pecuniary externality in other utility service territories where fixed costs are large and those fixed costs are recovered through volumetric retail prices. This combination of high fixed costs of electricity provision and low rooftop solar adoption currently describes much of the United States. Perhaps more importantly, many of these areas have never been the subject of empirical analysis of the determinants of uptake due to low penetration.

Our ACE modeling approach demonstrates that the equity implications of rooftop solar adoption arise, not because high-income utility customers are more likely to adopt, but because adoption creates a pecuniary externality that impacts LMI customers. This insight suggests several ways the ACE model can be adapted to explore other utility service areas or investigate the effect of future policy and technology changes on the pecuniary externality. Future work could incorporate a more complex customer agent that makes decisions about the size of the PV system, the size of coupled batteries, and battery dispatch decisions. This extension would allow the ACE model to explore time-of-use rates, net energy metering, and two-part tariffs that are currently being considered in many parts of the country. The spread of smart meters and other distributed energy technologies promises to provide the data necessary to characterize more complex agent behavior in future ACE models. Extending the utility agent to make investment decisions that change its energy mix and expand the grid would also be an exciting area for future work. Current industry models (e.g. EnCompass, Aurora) designed to consider generation and transmission investments do not consider how those investments impact customer behavior through prices.

## Methods

### Agent-based computational economic model of solar adoption

The structure of the ACE regulated power market consists of two general features: (i) economic agents that buy and sell electric power and (ii) generation and transmission assets that define the configuration of the grid network and determines how agent decisions impact other agents. The model is consistent with a regulated electricity market where a single wholesale utility agent generates and transmits electricity to satisfy electricity demand at least cost subject to (i) retail utility demand schedules for each customer type, (ii) individual generator marginal cost and generation constraints, (iii) capacity constraints and reactance of transmission lines, and (iv) node constraints that ensure supply equals demand. Economic agents include (i) a single regulated wholesale utility that generates power and sells it to retail utilities, (ii) retail utilities that purchase power from the wholesale utility agent and sell that power to end-use customers, and (iii) customer agents that purchase power from the retail utilities and choose when to adopt rooftop solar.

The utility agent chooses dispatch at each generating unit to minimize the total system cost according to a DC optimal power flow (OPF) model. A “zero-profit condition” is used to calculate a wholesale electricity price based on these cost-minimizing dispatch decisions. TVA’s least-cost planning program, mandated by Congress, results in it being operated similar to other regulated electricity markets. The retail utility agents choose retail electricity prices that apply to their service areas. Retail prices are determined by a Ramsey pricing algorithm (36) that accounts for wholesale prices and a grid access fee charged by the wholesale utility agent and the retail utility’s fixed costs.

Each customer agent chooses when to adopt a rooftop solar-plus-storage system that would completely satisfy their demand. The adoption decision is based on (i) the upfront cost of the system, (ii) expected energy bill savings from adoption, and (iii) carbon emissions offset by adoption. Expected energy bill savings from adoption depend on retail prices set by their respective retail utility which depend on the wholesale rates set by the wholesale utility agent. Carbon emissions offset by distributed solar adoption is determined by the dispatch decisions of the wholesale utility agent.

Many analyses of electricity markets estimate aggregate supply and demand curves (i.e. a top-down approach). In contrast, our ACE model is a bottom-up approach in that we specify the electricity demand of each customer agent type and the generation costs at each individual generating unit which are then aggregated up to reveal system-wide demand and supply curves. The model can be applied to most regulated utility markets. A general exposition of the model is provided in Appendix  Section 1 and Appendix  Table 1. Data and parameterization details are provided in Appendix  Section 2, Appendix Tables 2–11, and Appendix  Figures 1–5.

Our ACE model extends the AMES wholesale power market framework (37) in several ways. First and foremost, customer agents respond to changes in electricity prices by adopting rooftop PV according to adoption rules based on results of a choice experiment. Second, we consider a single generation utility agent which is more indicative of regulated electricity markets instead of the deregulated markets considered in AMES. Third, it includes a sub-module that computes regulated retail electricity prices (through Ramsey pricing) instead of assuming these prices are the result of an auction.

### Generation and transmission data

For each of TVA’s 141 generating units, we know the type of fuel used, generation constraints (e.g. min/max run times, ramping constraints, maximum capacities), and CO_2_ emissions. Generator characteristics come from the EIA-923, which is a survey that collects detailed data from electric power plants. CO_2_ emissions come from the EPA’s continuous emission monitoring system (CEMS).

We use PSSe simulation data provided by TVA to determine the total maximum transmission line capacity flowing between TVA’s 16 Customer Service Districts. The raw data from TVA contain ∼1,870 transmission lines that connect 5,940 buses. However, results from the choice experiment survey are not available on all 5,940 buses. To consolidate the buses and transmission lines into a smaller grid that is consistent with a reasonable scale for a choice experiment, the following series of steps were performed. First, buses were aggregated into TVA’s 16 Customer Service Districts. Second, the capacities of the transmission lines connecting the combined nodes were summed. Third, the reactance for each aggregated transmission line was calibrated to ensure the calculated generator commitments match the actual commitments from historical data.

### Electricity demand data

Hourly electricity demand is extremely inelastic; consumers do not alter their electricity usage based on the marginal cost of electricity generation, and retail utilities typically do not alter per-kWh electric rates based on the marginal cost of generation. We model inelastic hourly electricity demand by assuming a fixed quantity demanded, meaning the hourly demand curve is vertical for each hour of each day. To calculate the short-run electricity demand in each retail utility we first gather historic hourly electric load data for the entire TVA region from FERC form 714. We use 2019 data to construct representative hourly electricity demand for each retail utility in the region by dividing TVA load to each retail utility proportionally by population.

### Electricity prices

Consistent with electricity pricing in the TVA service area, retail utilities are charged a per-unit price and a fixed grid access fee while end-use customers are charged only a per-unit price. A critical component of our model is that per-unit prices and the grid access fee both adjust in response to model variables. Wholesale rates are equivalent to the wholesale utility’s average variable cost of electricity production. We input annual electricity production in MWh into a total variable cost equation for each generating unit to calculate total annual variable costs, and sum each unit’s costs to calculate total variable costs for the entire wholesale utility. We then divide annual total variable costs by the wholesale utility’s total annual production in MWh to calculate the initial wholesale price. Wholesale rates are updated after each annual model run based on simulated production from each generating unit.

Retail prices are determined based on the Ramsey pricing algorithm described in detail in the Appendix. To initialize retail prices in the Ramsey pricing algorithm, we gather data from electricity sales for end-use regional electric utilities from EIA 861 Annual Electric Power Industry Reports. Form 861 data includes customer counts for residential, commercial, and industrial electricity consumers, along with electricity usage and revenues generated from each consumer group.

Another key input in the Ramsey pricing algorithm is the fixed grid access fee charged by each retail utility, which we calculate directly from EIA 861 data. Recall that we assume each utility is restricted to earning zero profits (Revenue−Variable Cost−Fixed Cost=0), which is equivalent to Fixed Cost=Revenue−Variable Cost. We calculate fixed cost for each retail utility using total annual revenue for each consumer group and total annual electricity consumption for each consumer group reported directly in EIA 861 and our estimate for wholesale prices.

### Solar adoption and choice experiment

To specify the initial amount of solar adoption in each retail utility, we aggregated the DeepSolar data to obtain both counts and sizes of PV panels by Census Block Group (8). DeepSolar is a machine learning framework that analyzes satellite imagery to identify the GPS locations and sizes of PV panels. We then assigned these Census Block Groups to the retail utilities to obtain an estimate of the initial heterogeneity in solar deployment at different locations on our modeled grid.

To create solar adoption rules that vary across all customer agents, we conducted a survey of 2,307 households in the TVA region. Our study was approved by the Institutional Review Board of the University of Tennessee, Knoxville (IRB-19-05350-XM, 2019 October 30), and informed consent was obtained from all participants. The survey consisted of around 40 questions and took respondents about 15 min to complete. The survey included a stated-preference experiment, which presented six choice experiments each comparing three hypothetical solar arrays that vary in three dimensions: cost, electricity savings, and carbon emissions abatement. Finally, respondents answered a handful of socio-demographic questions. Respondents were collected through a Qualtrics panel sampled at the zip code level, so the number of respondents in each zip code roughly matches the distribution of households across the TVA region. Sampling at the zip code level allows us to precisely estimate solar adoption rules for high- and low-income agents with different price, electricity expenditure savings, and emission reductions sensitivities across each electrical bus. Details for the choice experiment survey are presented in Bostick et al. (38) and in the Appendix.

### Model validation

We validated our model at the individual agent behavior level and at the system-wide level. We compared the results of our OPF to the results of a similar OPF used by TVA for long-run planning purposes. We also validated residential solar adoption behavior in our model by comparing our basecase simulation results to a 2050 forecast of distributed solar adoption in the TVA service area conducted by EPRI. Finally, we compared retail utility electricity prices to retail electricity price data in the TVA service area. See Appendix  Section 3 for additional details.

## Supplementary Material

pgaf356_Supplementary_Data

## Data Availability

All data in this study are publicly available or from existing peer-reviewed studies. All data and code used in this analysis is available for download from https://github.com/gassergalalali/DER-TVA.
