# Supplementary material for: The equity implications of pecuniary externalities on an electric grid
Source: PNAS Nexus. 2025 Dec 9;4(12):pgaf356. doi: 10.1093/pnasnexus/pgaf356 (PMC12687347; doi:10.1093/pnasnexus/pgaf356)
Supplement: pgaf356_Supplementary_Data [file pgaf356_supplementary_data.pdf]

Appendix for: The equity implications of pecuniary externalities on an electric grid

Charles Sims * Gasser G. Ali †

J. Scott Holladay ‡ Tim Roberson § Chien-fei Chen ¶ Islam H. El-adaway ‖

October 31, 2025

*TVA Distinguished Professor of Energy and Environmental Policy, Baker School of Public Policy and Department of Economics, University of Tennessee, Knoxville

†Assistant Professor, Department of Civil Engineering, The University of Texas Rio Grande valley

‡Associate Professor, Department of Economics, University of Tennessee, Knoxville

§Assistant Professor, Department of Economics, Finance, and Marketing, Tennessee Tech University

¶Professor, Department of Sociology, Anthropology, Criminal Justice, Clemson University

‖Hurst-McCarthy Professor of Construction Engineering and Management, Professor of Civil Engineering, and Founding Director of Missouri Consortium of Construction Innovation, Department of Civil, Architectural, and Environmental Engineering/Department of Engineering Management and Systems Engineering, Missouri University of Science and Technology

Appendix Table 1: Notation used in the ACE model

Category	Notation	Definition
Time	$H \in [0, ..., 23]$	hours
	$t \in [0, ..., 30]$	years
Electricity generation	$i \in N$	generators
	$\bar{q}_i$	nameplate capacity of generator i
	$MC_i = a_i + 2b_i q_i(H)$	marginal cost for generator i in hour H
	$q_i(H)$	quantity supplied by generator i in hour H
Retail utilities	$j \in J$	Retail utilities
	$z \in Z$	customer type
	$q_j(H)$	total hourly demand in retail utility j
	c_{jz}, d_{jz}	long-run demand parameters type z, retail utility j
	I_{jz}	number of customers type z, retail utility j
	$D_{jz}(H)$	per-capita hourly demand type z, retail utility j
	$Z_{jz,t}$	average annual demand type z, retail utility j
Transmission Grid	$k \in K$	nodes on the grid
	$x \in X$	transmission lines connecting nodes
	m	an arbitrary node
	line km	a line connecting node k to m
	θ	penalty constant in OPF
	δ_k	phase angle at node k
	q_{km}	power flow over line km
	$q_{km,max}$	transmission line km constraint
	$Reactance_{km}$	reactance on line km
	$Capacity_{km}$	line capacity km
Regulation and pricing	w	wholesale electricity price per-unit
	Q_{jz}	annual demand type z retail utility j
	TVC	utility annual variable cost
	F	utility annual fixed cost
	$Profit$	utility annual profit constraint
	p_{jz}	retail price for group z, retail utility j
	f_j	annual fixed cost for retail utility j
	λ	lagrangian multiplier for profit constraint
	ϵ_{jz}	demand elasticity group z, retail utility j
Customer Agent Solar Adoption	$Pr_{jz,t}$	distributed solar adoption probability
	$savings_{jz,t}$	electricity bill savings type z retail utility j
	$abatement_{jz,t}$	CO2 emissions avoided time t
	e_t	emissions intensity of electricity generation
	$cost_t$	upfront PV system cost
	S_{jz}	quantity of PV generation in retail utility j
Dispatch (Supply) Curve	u	primary fuel type
	v	prime mover
	τ	time (month)
	$MW_{uv,\tau}$	monthly output for fuel type u prime mover v
	$TVC_{uv,\tau} = a_1 MW_{uv,\tau} + a_2 MW^2_{uv,\tau} + \epsilon_{uv,\tau}$	total variable cost estimate in month τ
	$Consumption_{uv,\tau}$	fuel consumption
	$HR_{uv,\tau}$	heat rate for fuel type u prime mover v
	$Heat_{uv,\tau}$	average heat content for fuel type u prime mover v
	$NetGen_{uv,\tau}$	total output class for fuel type u prime mover v
	$Fuel_{u,\tau}$	average fuel cost
	Cap_{uv}	capacity factor for fuel type u prime mover v

1 Agent-Based Computational Economic Model of Solar Adoption

A notation guide is provided in Appendix Table 1. The grid components include 1) K nodes or buses which represent different locations in the grid such as towns or cities, 2) N generating units, owned and operated by the wholesale utility, and 3) X transmission lines that connect the nodes and transmit power between them. Each generating unit i is characterized by a marginal cost of generation and generation constraints that vary based on fuel type and technology. Each node k is connected to at least one other node through a transmission line. The distance of a transmission line coupled with the transmission capacities restrictions and reactances on that line determine the cost of transmitting electricity from the point where it is generated to a retail utility that sells the electricity to their customers.

Each agent makes decisions based on the behavior of other agents and their own private information. The single regulated wholesale utility agent makes three decisions. First, the utility agent chooses hourly dispatch at each generating unit to meet total demand at minimum cost based on locational marginal prices (LMP) that are private information for the wholesale utility. LMPs reflect the cost of meeting electricity demand (load) at different locations, accounting for the patterns of demand, generation, and the physical limits of the transmission system. Hourly dispatch and LMPs are the solution to a DC optimal power flow (OPF) problem that operates on an hourly time step H=0, 1,..., 23 to capture electricity demand variation during the day with identical hourly demand profiles each day. OPF is an hourly optimization problem where the objective is to minimize the cost of electricity generation subject to equality constraints that balance supply and demand at each node in the transmission network and inequality constraints that reflect network operating limits (line flows, generation capacities, reactances, etc.) and limits on management actions. Second, the utility agent also sets a single wholesale electricity price based on the total variable costs of generation and transmission implied from its dispatch decisions and resulting LMPs produced from the OPF. Third, the regulated utility sets a grid access fee to cover the fixed cost of generation and transmission assets as well as a regulated profit requirement.

The single regulated wholesale utility produces and transmits electricity to the J retail utilities. Each retail utility then sells and distributes electricity to local retail customers. Each retail utility serves $Z = 4$ customer agent types (indexed by z): industrial, commercial, LMI residential, and high-income residential. Each customer agent is characterized by perfectly inelastic short-run demand that varies by hour. Retail utilities set retail electricity prices based on the wholesale price set by the wholesale utility agent and their own fixed costs. The retail utilities charge customers a constant price for each kwh regardless of when it is

consumed. This constant retail price, which most closely reflects current practice throughout most of the country, is calculated within the ACE model following Ramsey pricing which marks up the wholesale price to account for the retail utility's fixed cost of distribution. This single retail price is adjusted every 6 months in response to wholesale prices determined by the wholesale utility agent.

There is no entry of traders into or exit of traders from the regulated market. The wholesale utility is allowed to go into debt without penalty or forced to exit. The model also assumes no system disturbances or shocks that would void the financial contracts determined on each day for the day-ahead market. This implies a real-time market is unnecessary.

Each customer agent chooses when to adopt a rooftop solar plus storage system that would completely satisfy their demand. The adoption decision is based on 1) the upfront cost of the system, 2) expected energy bill savings from adoption, and 3) carbon emissions offset by their adoption. Expected energy bill savings from adoption depend on retail prices set by their respective retail utility. Carbon emissions offset by distributed solar adoption is determined by the dispatch decisions of the utility agent. Electricity produced by the PV system does not flow back to the grid.

1.1 Solar adoption and pecuniary externalities

Customer agent type z in retail utility j can purchase electricity from their retail utility at price p_{jz} or switch to a rooftop PV system which requires an upfront, fixed cost investment but no marginal cost. Customers base their adoption decision on three factors: 1) the upfront cost of the system, 2) the future energy bill savings, and 3) environmental implications of their PV adoption decision. Customers only consider battery plus solar systems such that distributed solar adoption manifests as a reduction in customers instead of a change in the hourly load curve. Specifically, the number of customer type z in retail utility j in year $t+1$ is

$$I_{jz,t+1} = (1 - Pr_{jz,t}(cost_t, savings_{jz,t}, abatement_{jz,t})) * I_{jz,t} \tag{1}$$

where $I_{jz,t}$ is the population of customer type z in retail utility j in year t

$$Pr[adopt]_{jz,t} = \pi_{0,jz} + \pi_{1,jz} cost_t + \pi_{2,jz} savings_{jz,t} + \pi_{3,jz} abatement_{jz,t} \tag{2}$$

is the probability of solar adoption and

$$AR_{jz} = \frac{I_{jz,T} - I_{jz,0}}{I_{jz,0}} \tag{3}$$

is the adoption rate over T years for customer type z in retail utility j.

The probability of solar adoption varies by retail utility and customer type and depends on the electricity bill savings ($savings_{jz,t}$), the pollution emissions avoided from adopting solar ($abatement_{jz,t}$), and the upfront cost of the PV system ($cost_t$). We hypothesize that lower PV system costs and higher electricity bill savings will increase the probability of adoption for financial reasons: $\pi_{1,jz} < 0$ and $\pi_{2,jz} > 0$. Electricity bill savings are determined within the model according to $savings_{jz,t} = p_{j,t}S_{jz}$ where S_{jz} is the annual quantity of electricity generated from a solar system by customer type z in retail utility j. To allow cost to be tied to world solar panel price trends, $cost_t$ is exogenous to the model. Emissions abatement is also endogenous to the model since the emission intensity of grid electricity evolves as the utility agent adjusts generation in response to the lower demand triggered by solar adoption. We hypothesize that more emission reductions will increase the probability of adoption: $\pi_{3,jz} > 0$.

Solar adoption can generate pecuniary externalities whereby adoption by one customer type raises retail rates for another customer type:

$$\left[\frac{\partial p_{jz'}}{\partial p_{jz}} + \frac{\partial p_{j'z'}}{\partial p_{j'z}}\frac{\partial p_{j'z}}{\partial p_{jz}}\right]\frac{\partial p_{jz}}{\partial I_{jz}} \tag{4}$$

When $\frac{\partial p_{jz}}{\partial I_{jz}} < 0$, solar adoption results in higher retail rates for the same customer group. This is the "utility death spiral" result in which solar adoption begets more adoption through adjustments in retail prices. The sign and magnitude of this term depends on the size of utility fixed costs, how those fixed costs are passed on to customer agents, and how customer agents adjust their solar adoption decision in response to higher electricity rates. If fixed costs are sufficiently low, solar adoption could lower retail rates by reducing generation costs and transmission congestion. However, if fixed costs are large, solar adoption will raise retail rates as those fixed costs must be spread over a smaller group of customers.

Conditional on this term being negative, distributional concerns about pecuniary externalities between customer groups arise when the terms in brackets are positive. When the first term in brackets is positive, solar adoption by customer type z results in higher retail rates for customer type z' in the same retail utility. When the second term in brackets is positive, the pecuniary externality spreads across retail utilities. For example, solar adoption by high-income residential customers in an urban retail utilities raises retail rates for low-income residential customers in urban and rural retail utilities. As we will show, the sign and magnitude of these partial derivatives depends on the retail rate structure, how sensitive customers are to electricity bill

savings when making solar adoption decisions $\pi_{1,jz}$, the transmission network, and the wholesale electricity rate structure.

The pecuniary externality raises electric bills but it also has a potential upside. If customer solar adoption is sufficiently sensitive to energy bill savings, the pecuniary externality will incentivize more solar adoption. When you combine the pecuniary externality with the utility death spiral effect, PV system adoption by one customer type incentivizes adoption by another customer type.

The pecuniary externality's impact on solar adoption has market efficiency and equity implications. By incentivizing adoption, the pecuniary externality counteracts the incentive to free-ride as others adopt solar and reduce pollution emissions from the electricity sector. Indeed this free-riding effect is also in our model and works through the $abatement_{jz,t}$ variable. The equity implications depend on two factors. The first is whether the solar adoption rate AR_{jz} differs across customer types (i.e., an adoption gap). The second is whether the change in the solar adoption rate due to the pecuniary externality, ΔAR_{jz}, differs across customer types (i.e., death spiral gap). For example, high-income solar adoption may be less sensitive to changes in electric bills than LMI customers since high-income customers have a lower energy burden.

1.2 A bottom-up system of supply and demand

Electricity is supplied by N dispatchable generators owned and operated by the regulated utility agent. Each generator is characterized by nameplate capacity $\bar{q}_i$ (in MW), CO2 emissions intensity (Tons/MWh), and the unit's marginal cost of supplying electricity. The marginal cost for generator i for use in every hour H is

$$MC_i = a_i + 2b_i q_i(H) \tag{5}$$

where $0 \leq q_i(H) \leq \bar{q}_i$, in MWs, is the quantity of electricity supplied by generator i and MC_i is cost per MWh for the electricity supply $q_i(H)$. The parameters a_i ($\$/MWh$) and b_i ($\$/MWh^2$) are nonnegative and depend on fuel costs and generation technology. The supply (dispatch) curve is built from the bottom up aggregating the marginal cost curves for the N generating units.

Each retail utility is comprised of I_j customers which represents the sum of low-income residential, high-income residential, commercial, and industrial customers: $I_j = \sum_z I_{jz}$ where I_{jz} is the number of customer agent type z in retail utility j. Following a large literature that shows customers are insensitive to changes in electricity rates, all customer agents in the model have perfectly inelastic hourly demand. We do not treat customer's long-run yearly electricity demand as perfectly inelastic, but rather estimate elasticities

of demand for each consumer group over the long-run, as described in the Ramsey pricing section of the paper. The assumption of inelastic short-run demand and more elastic long-run demand is consistent with consumers being unable to adjust electricity consumption in response to prices on an hour-to-hour basis, but being able to change habits and make investments in energy efficiency in the long-run (Zhu et al. 2018).

For each hour H, the quantity purchased (in MWs) in retail utility j is

$$q_j(H) = \sum_z I_{jz} D_{jz}(H) \tag{6}$$

where $0 \leq D_{jz}(H)$ is the per capita hourly demand of customer type z in retail utility j.

1.3 Transmission and locational marginal prices

The transmission network is composed of X transmission lines that link the K nodes on the grid. Each line accounts for temperature and voltage restrictions that constrain the utility's ability to balance demand (load) and generation. Our model operates over a user-specified AC transmission grid characterized by the reactance and maximum capacity of each transmission line km that flows power from node k to node m.

The wholesale utility uses retail utility fixed demands, the marginal cost and generation constraints at each generating unit, and the transmission constraints as input data for a DC optimal power flow (OPF) problem. The OPF problem solver is based on the augmented DC-OPF problem proposed by Sun and Tesfatsion (2010). The standard AC-OPF problem minimizes the total variable generation costs subject to nonlinear balance, branch flow, and production constraints for real and reactive power. AC-OPF problems are often approximated by a more tractable DC-OPF problem that focuses exclusively on real power and utilizes linearized power constraints. We then augment the standard DC-OPF objective function with a penalty function on the sum of the squared voltage angle differences. This augmentation transforms the DC-OPF problem into a *strictly* convex quadratic programming problem.

The augmented DC-OPF problem can be used to generate solutions for the LMPs, phase angles at the nodes, and real power flows along each transmission line. The solution to the OPF problem is a series of hourly dispatch and LMPs that minimize the utility's total variable cost (TVC) of meeting a given load profile:

$$\min_{(q_1,\ldots,q_N)} TVC = \sum_i^N [a_i q_i(H) + b_i q_i^2(H)] + \theta \sum_x^X [\delta_k - \delta_m]^2 \tag{7}$$

subject to node balance constraints for each node k

$$\sum_j [q_j(H)] - \sum_i [q_i(H)] + \sum_x [q_{km}] = 0 \quad (8)$$

generation constraints for each generator $0 \leq q_i(H) \leq \bar{q}_i$, transmission line constraints for each transmission line km $|q_{km}| \leq q_{km,max}$ where θ is a penalty constant, δ_k is the phase angle at node k, q_{km} is the power flow in the transmission line from node k to node m. The results of the optimization problem, produced using dual stage optimization (Goldfarb and Idnani, 1983), are the commitment of each generator q_i, the power flow at each transmission line q_{km}, and the LMP at each node LMP_k.

The problem is a positive definite quadratic problem subject to linear equality and inequality constraints. The dual stage optimization method relies on the unconstrained minimum of the objective function as the starting point and utilizes Cholesky and QR factorizations to update the minimum of the objective function. It is efficient and numerically stable and has been used to successfully solve other DC-OPF problems (Sun and Tesfatsion, 2007). The results are then used to calculate wholesale and retail prices that are fed back to the agents in the ACE model. The problem is solved for every hour in the simulation.

1.4 Wholesale and retail electricity rates

The wholesale and retail utilities will change the wholesale and retail electricity prices to reflect changes in production cost and electricity demand as customers adopt rooftop solar. Adoption of rooftop solar will lower the marginal cost of producing electricity, which will lower retail electricity prices all else equal. However, adoption of rooftop solar will not lower the fixed costs of production, transmission, and distribution networks. If many customers adopt rooftop solar, these fixed costs will need to be recovered through sales to fewer consumers, necessitating increases in electricity prices to customers who have not adopted rooftop solar. If electricity prices rise, then customers sensitive to potential electricity bill savings will adopt rooftop solar. This may result in additional electricity price increases, causing a feedback loop of higher prices and increased adoption. To determine whether adoption of rooftop solar raises or lowers electricity prices, we develop a model of dynamic price adjustment.

The wholesale utility charges a single uniform wholesale price w for each kilowatt/hour of electricity sold. While LMPs measure the marginal cost of producing and transmitting electricity to each node on the transmission grid, utilities generally charge the same per-kilowatt/hour wholesale price regardless of where a customer is located on the grid.

To see why this is an important distinction, consider a situation in which the wholesale utility serves

two retail utilities: A and B. A large, low-cost nuclear generator provides all electricity in retail utility A, while demand in retail utility B is served by more expensive coal-powered generators. LMPs in retail utility B will be higher than in retail utility A, but this does not mean that customers in retail utility B will be charged higher electric prices. The wholesale utility serving the region encompassing retail utilities A and B will instead charge the same per-unit wholesale price for electricity to each retail utility, and each retail utility will pass the wholesale price through to retail customers.

The wholesale utility is regulated to make a certain maximum annual profit. To satisfy the maximum annual profit constraint, the utility's total annual revenue must be less than or equal to the total cost of producing electricity in that year plus the allowed profit $Profit$

$$\sum_{j=1}^{J} wQ_j - \frac{365 \sum_H TVC}{\sum_{j=1}^{J} Q_j} - F \leq Profit \tag{9}$$

where $Q_j = 365 \sum_H q_j(H)$ is annual demand for retail utility j, $\frac{365 \sum_H TVC}{\sum_{j=1}^{J} Q_j}$ is the utility's average annual variable cost of producing electricity, and F is the utility's annual fixed cost. Fixed costs, any cost which does not increase with the amount of electricity produced and sold, include the cost of maintaining generators and transmission lines, salaries for employees, the cost of maintaining information technology services, and the cost of debt service. Consistent with wholesale electricity pricing in many parts of the U.S., we assume the utility passes through its annual fixed cost and profit requirement $F + Profit$ to retail utilities as an annual fixed grid access fee. The grid access fee implies the wholesale utility must charge a wholesale price w to cover the annual variable cost of electricity production, $w = \frac{365 \sum_H TVC}{(\sum_{j=1}^{J} Q_j)^2}$. This approach is similar to TVA's 2018 wholesale rate changes, which imposed a fixed grid access fee, designed to be revenue neutral when accounting for per-unit energy fees.

Each retail utility purchases electricity from the wholesale utility at rate w, and pays an annual grid access fee proportional to $F + Profit$ to cover the wholesale utility's fixed costs. Retail prices are set by each retail utility based on these wholesale prices, consumer demand for electricity, and the retail utility's own cost of doing business. In practice, rate setting is a long-term, legally involved process in which both the wholesale utility and each retail utility must justify rate changes to regulators and stakeholders. We simplify this process into a single system of equations based on a Ramsey pricing model (Berry 2000), which determines per-kilowatt/hour retail electricity prices p_{jz} for $z \in Z$ customer groups in each retail utility.

Ramsey (1927) first determined optimal prices for a regulated monopolist selling to multiple consumer groups, and this result was later expanded upon in Boiteux (1956). The Ramsey-Boiteux pricing solution, or Ramsey pricing for short, determines the welfare maximizing prices for a regulated monopolist (such

as a utility) as a function of the elasticity of demand for each consumer group and the marginal and fixed production costs to the monopolist. The Ramsey pricing module calculates the consumer welfare-maximizing electric rates that each retail utility will charge to customers. These rates are determined as a function of four parameters: the long-run customer elasticities of demand for electricity, the wholesale price of electricity, the retail utility's fixed cost of selling electricity, and the number of customers in each group z in each retail utility. This approach implies the retail utility sets prices that maximize total social welfare subject to earning enough revenue to cover their own fixed costs. The Ramsey optimization problem for retail utility j can be written in the form of the following maximization problem

$$\max_{p_{jz}} \sum_{z}^{Z} \int_{0}^{Q_{jz}(p_{jz})} p_{jz}(Q)dQ - w \sum_{z=1}^{Z} Q_{jz} - f_j \tag{10}$$

subject to a similar profit condition facing the wholesale utility:

$$\lambda : \qquad \sum_{z}^{Z} p_{jz}(Q_{jz}) * Q_{jz} - w \sum_{z=1}^{Z} Q_{jz} - f_j = 0 \tag{11}$$

where $Q_{jz} = 365 \sum_H I_{jz} D_{jz}(H)$, λ is a Lagrangian multiplier, and f_j is annual fixed cost for retail utility j, which includes the portion of fixed costs passed on by the wholesale utility as a grid access fee, and the retail utility's own overhead costs unrelated to the cost of purchasing electricity from the wholesale utility.

Social welfare is the sum of consumer plus producer surplus, which is the total area under the demand curve for each group in the retail utility less the retail utility's total cost of supplying electricity to all customers $w \sum_{z=1}^{Z} Q_{jz} + f_j$. Solving this problem yields the following equation defining the welfare maximizing prices for each customer group in each retail utility:

$$\frac{p_{jz} - w}{p_{jz}} = \frac{\frac{\lambda}{1+\lambda}}{\epsilon_{jz}} \tag{12}$$

where $\epsilon_{jz}$is the long-run price elasticity of demand for electricity of group z. In words, this equation relates the social welfare maximizing per-unit electricity prices to wholesale costs, consumer demand parameters, and the effects of the regulated profit constraint.

The ratio of markups for any two groups z, z' does not depend on λ

$$\frac{\frac{p_{jz}-w}{p_{jz}}}{\frac{p_{jz'}-w}{p_{jz'}}} = \frac{\epsilon_{jz'}}{\epsilon_{jz}} \tag{13}$$

allowing us to write the Ramsey-optimal price for every consumer group in terms of the price charged to a

single reference group

$$p_{jz} = \frac{w}{1 - \frac{\epsilon_{jz'}}{\epsilon_{jz}}(1 - \frac{w}{p_{jz'}})} \tag{14}$$

Based on equation 14 and 11, equilibrium prices are fully characterized by $(w, f_j, \epsilon_{jz}, \epsilon_{jz'})$ for each retail utility $j \in J$ and each consumer group $z \in Z$, where $\epsilon_{jz'}$ is the elasticity of demand for a reference group. We estimate the per-unit wholesale electric rate w and ϵ_{jz} for each consumer group to recover the relative equilibrium prices for each consumer group from equation 13 or 14. To recover prices for each retail utility, we find the relative prices which ensure that each retail utility satisfies the zero profit constraint. This requires us to estimate the annual fixed costs f_j for each retail utility. If we know the fixed costs, we can calculate the reference price from equation 14 necessary to ensure the zero profit constraint binds.

Electricity prices in each retail utility update as consumers adopt distributed solar and defect from the electric grid. We describe the process used for updating retail prices in the following series of steps:

0. Calculate $e_{jz}, e_{jz'}, w$ and f_j from historic data, cost estimates, and demand estimates as described in the following section.

1. Initialize the agent-based model using historic prices for each customer group in each retail utility. Note that prices will vary by retail utility, year and consumer group (residential, commercial, and industrial) in this model, but will not vary for residential consumers of different income groups.

2. After one six month simulation run, re-calculate the wholesale electric rate w based on simulated production from each generator. Use w to calculate new prices for each consumer group.

3. Use the new w and I_{jz} to recalculate the retail prices, based on equation 14 and the zero profit constraint $\sum_z^Z p_{jz}(p_{jz'}) * Q_{jz} - w \sum_{z=1}^Z Q_{jz} - f_j = 0$. Return to step 2.

This process creates a tension between two effects. First, distributed solar adoption decreases the variable costs of electricity production, which creates downward pressure on retail electricity rates. However, distributed solar adoption decreases electricity supplied by the utility agent, meaning that fixed cost pass-through is divided across fewer kilowatts, creating upward pressure on electricity rates. Our model determines which of these effects dominates, the path of retail electricity prices over time to different customer agents, and the magnitude of the pecuniary externality between customer agents.

1.5 Discussion of Pricing Strategies

Our pricing model assumes that retail consumers pay a flat volumetric price for electricity which covers both the fixed and variable costs of electricity production, creating the dynamic where prices may increase to cover fixed costs. However, alternative pricing strategies could create different pricing dynamics as consumers adopt solar. For instance, a two-part tariff pricing strategy would cover fixed costs with fixed, lump-sum access fees in retail consumers' bills while volumetric fees cover only variable costs of electricity production. A two-part tariff would therefore not result in rising retail prices to cover fixed costs if consumers adopt solar.

We believe that a flat volumetric price is a reasonable assumption for two reasons. First, our model assumes that consumers who adopt solar defect from the grid entirely. This leaves fewer consumers to cover utility fixed costs, meaning that even with a two-part tariff, fixed fees would need to rise for the remaining non-adopting consumers. If grid defection is an accurate assumption, then any pricing strategy would require that more costs be covered by the remaining non-adopting consumers, giving them an incentive to defect from the grid by adopting solar. Grid defection is a good model for the current technological reality, in which home battery storage systems allow consumers to store solar power for use at non-peak times.

Secondly, although two-part tariffs have theoretical advantages over flat volumetric rates, in practice most retail utilities' pricing is much closer to a volumetric rate than to covering all fixed costs with fixed access fees. The TVA reports that approximately 67% of their costs are fixed, and retail utilities add their own fixed costs. For a fixed access fee to truly cover all fixed costs, it would need to amount to over 70% of consumers' electricity bills. This would mean, for instance, that a $200 monthly electricity bill would need to include a fixed charge of over $140. To our knowledge, there are no retail utilities that adopt pricing strategies with such large fixed fees.

2 Data and Model Parameterization

2.1 Generator characteristics

All generator characteristic used in the ACE model are presented in Appendix Table 2. Using monthly data EIA-923 data from 2008 to 2017, costs are calculated by multiplying the rate of fuel use by the cost of input fuel:

$$TVC_{uv,\tau} = Fuel_{u,\tau} * HR_{uv,\tau} * MW_{uv,\tau} \tag{15}$$

where $Fuel_{u,\tau}$ is the average fuel cost ($ per MMBtu) differentiated by fuel type and weighted by the quantity of fuel received,

$$HR_{uv,\tau} = [\frac{Consumption_{uv,\tau} * Heat_{uv,\tau}}{NetGen_{uv,\tau}}] \tag{16}$$

is the heat rate or the input energy used per MW per hour, $Consumption$ is the quantity consumed in physical units for electric generation by fuel type (e.g., mcf for natural gas, short tons for coal, and barrels for oil), $Heat$ (MMBtu per physical unit) is the average heat content for TVA plants with the same fuel type and prime mover, and $NetGen$ (MWh) is the aggregate of electrical output for TVA plants within the same fuel type and prime mover group. Lastly, MW is calcuated as:

$$MW_{uvt} = \frac{NetGen_{uvt}}{30.42 * 24 * Cap_{uv}} \tag{17}$$

where Cap_{uv} is the capacity factor which is the percentage of time that a generator is operating (i.e., the ratio of actual generation to potential generation) multiplied by 30.42 days times 24 hours to convert to MWh. Annual U.S. averages for capacity factor by fuel type and prime mover come from the Electric Power Monthly Report by EIA.

We also calculated TVA-specific capacity factors for fuel and prime mover combinations in which data was available which included steam turbines for coal (BIT and SUB) and for the combination of combined cycle combustion turbine (CT) and combined cycle steam (CA) for natural gas. Capacity factors were calculated by dividing the monthly net generation by a generator's nameplate capacity. An annual average capacity factor was calculated for TVA generators within each fuel type and prime mover group. Net generation data is from the EIA-923 while nameplate capacity data is from the EIA-860 survey, which collects characteristics of electric power plants.

TVC and MW are calculated consistently for all fuel types including coal, oil, and natural gas with the following exceptions. Given the integration between combined cycle steam (CA) and combined cycle combustion turbine (CT) technologies for natural gas, generation and consumption data was aggregated for these prime movers, and single coefficients are estimated for natural gas combined cycle turbines. Additionally, nuclear reactors are not required to report some EIA data such as fuel costs, heat content, and quantity consumed in physical units. Therefore, for nuclear reactors, national average heat rates from EIA-923 and weighted annual average prices from the EIA Uranium Marketing Annual Report are used to calculate costs. However, net generation for TVA-specific nuclear reactors is used to calculate MW.

Appendix Table 2: Generator characteristics.

#	Location	Plant Name	Type	Max Capacity (MW)	CO2 Emissions (Tons/MWh)
1	Memphis	Allen	Combined	547.67	0.3788
2	Memphis	Allen	Combined	547.67	0.3749
3	Memphis	Allen	Natural Gas	17.58	0.9700
4	Memphis	Allen	Natural Gas	17.58	0.9700
5	Memphis	Allen	Natural Gas	17.58	0.9700
6	Memphis	Allen	Natural Gas	17.58	0.9700
7	Memphis	Allen	Natural Gas	17.58	0.9700
8	Memphis	Allen	Natural Gas	17.58	0.9700
9	Memphis	Allen	Natural Gas	17.58	0.9700
10	Memphis	Allen	Natural Gas	17.58	0.9700
11	Memphis	Allen	Natural Gas	17.58	0.9700
12	Memphis	Allen	Natural Gas	17.58	0.9700
13	Memphis	Allen	Natural Gas	17.58	0.9700
14	Memphis	Allen	Natural Gas	17.58	0.9700
15	Memphis	Allen	Natural Gas	17.58	0.9700
16	Memphis	Allen	Natural Gas	17.58	0.9700
17	Memphis	Allen	Natural Gas	17.58	0.9700
18	Memphis	Allen	Natural Gas	17.58	0.9700
19	Memphis	Allen	Natural Gas	53.83	0.9414
20	Memphis	Allen	Natural Gas	53.83	1.2792
21	Memphis	Allen	Natural Gas	53.83	0.9413

Appendix Table 2: Generator characteristics.

#	Location	Plant Name	Type	Max Capacity (MW)	CO2 Emissions (Tons/MWh)
22	Memphis	Allen	Natural Gas	53.83	0.7180
23	Jackson	Gleason	Natural Gas	173.33	0.6350
24	Jackson	Gleason	Natural Gas	173.33	0.6355
25	Jackson	Gleason	Natural Gas	165	0.6426
26	Columbia	Johnsonville	Natural Gas	56.00	0.7122
27	Columbia	Johnsonville	Natural Gas	56.00	0.6914
28	Columbia	Johnsonville	Natural Gas	56.00	0.7500
29	Columbia	Johnsonville	Natural Gas	56.00	0.7799
30	Columbia	Johnsonville	Natural Gas	56.00	0.5387
31	Columbia	Johnsonville	Natural Gas	56.00	0.6336
32	Columbia	Johnsonville	Natural Gas	56.00	0.6520
33	Columbia	Johnsonville	Natural Gas	56.00	0.6931
34	Columbia	Johnsonville	Natural Gas	56.00	1.0357
35	Columbia	Johnsonville	Natural Gas	56.00	0.5163
36	Columbia	Johnsonville	Natural Gas	56.00	0.7100
37	Columbia	Johnsonville	Natural Gas	56.00	0.6989
38	Columbia	Johnsonville	Natural Gas	56.00	0.7256
39	Columbia	Johnsonville	Natural Gas	56.00	0.6865
40	Columbia	Johnsonville	Natural Gas	56.00	0.6913
41	Columbia	Johnsonville	Natural Gas	56.00	0.7419
42	Columbia	Johnsonville	Natural Gas	84.42	0.7014
43	Columbia	Johnsonville	Natural Gas	84.42	0.7087
44	Columbia	Johnsonville	Natural Gas	84.42	0.7343
45	Columbia	Johnsonville	Natural Gas	84.42	0.4414
46	Jackson	Lagoon Creek	Combined	272.42	0.3708
47	Jackson	Lagoon Creek	Combined	272.42	0.3854
48	Jackson	Lagoon Creek	Natural Gas	83.58	0.7435
49	Jackson	Lagoon Creek	Natural Gas	83.58	0.7122
50	Jackson	Lagoon Creek	Natural Gas	83.58	0.7081

Appendix Table 2: Generator characteristics.

#	Location	Plant Name	Type	Max Capacity (MW)	CO2 Emissions (Tons/MWh)
51	Jackson	Lagoon Creek	Natural Gas	83.58	0.7141
52	Jackson	Lagoon Creek	Natural Gas	83.58	0.7363
53	Jackson	Lagoon Creek	Natural Gas	83.58	0.7355
54	Jackson	Lagoon Creek	Natural Gas	83.58	0.7170
55	Jackson	Lagoon Creek	Natural Gas	83.58	0.7135
56	Jackson	Lagoon Creek	Natural Gas	82.42	0.7127
57	Jackson	Lagoon Creek	Natural Gas	82.42	0.7148
58	Jackson	Lagoon Creek	Natural Gas	82.42	0.7225
59	Jackson	Lagoon Creek	Natural Gas	82.42	0.7146
60	Jackson	Brownsville	Natural Gas	115.08	0.6896
61	Jackson	Brownsville	Natural Gas	115.08	0.6429
62	Jackson	Brownsville	Natural Gas	120.17	0.7195
63	Jackson	Brownsville	Natural Gas	120.17	0.7287
64	Nashville	Cumberland	Coal	1218.33	1.0393
65	Nashville	Cumberland	Coal	1218.33	1.0122
66	Murfreesboro	Gallatin	Coal	221.00	1.0347
67	Murfreesboro	Gallatin	Coal	221.00	1.0550
68	Murfreesboro	Gallatin	Coal	269.00	1.0628
69	Murfreesboro	Gallatin	Coal	264.00	1.0571
70	Murfreesboro	Gallatin	Natural Gas	79.17	0.8271
71	Murfreesboro	Gallatin	Natural Gas	79.17	0.8690
72	Murfreesboro	Gallatin	Natural Gas	79.17	0.7672
73	Murfreesboro	Gallatin	Natural Gas	79.17	0.8453
74	Murfreesboro	Gallatin	Natural Gas	83.17	0.7205
75	Murfreesboro	Gallatin	Natural Gas	83.17	0.7272
76	Murfreesboro	Gallatin	Natural Gas	83.17	0.7107
77	Murfreesboro	Gallatin	Natural Gas	83.17	0.7115
78	Muscle Shoal	Colbert	Natural Gas	52.67	0.9055
79	Muscle Shoal	Colbert	Natural Gas	52.67	0.9683

Appendix Table 2: Generator characteristics.

#	Location	Plant Name	Type	Max Capacity (MW)	CO2 Emissions (Tons/MWh)
80	Muscle Shoal	Colbert	Natural Gas	52.67	0.9154
81	Muscle Shoal	Colbert	Natural Gas	52.67	0.9230
82	Muscle Shoal	Colbert	Natural Gas	52.67	0.8883
83	Muscle Shoal	Colbert	Natural Gas	52.67	0.8921
84	Muscle Shoal	Colbert	Natural Gas	52.67	0.9154
85	Muscle Shoal	Colbert	Natural Gas	52.67	0.9154
86	Muscle Shoal	Browns Ferry	Nuclear	1112.17	-
87	Muscle Shoal	Browns Ferry	Nuclear	1116.00	-
88	Muscle Shoal	Browns Ferry	Nuclear	1116.92	-
89	Chattanooga	Sequoyah	Nuclear	1159.92	-
90	Chattanooga	Sequoyah	Nuclear	1147.00	-
91	Chattanooga	Watts Bar	Nuclear	1158.08	-
92	Chattanooga	Watts Bar	Nuclear	1164.58	-
93	Knoxville	Kingston	Coal	120.00	1.1395
94	Knoxville	Kingston	Coal	116.00	1.1349
95	Knoxville	Kingston	Coal	116.00	1.1371
96	Knoxville	Kingston	Coal	120.00	1.1357
97	Knoxville	Kingston	Coal	161.00	1.1268
98	Knoxville	Kingston	Coal	161.00	1.1386
99	Knoxville	Kingston	Coal	161.00	1.1390
100	Knoxville	Kingston	Coal	161.00	1.1351
101	Knoxville	Kingston	Coal	161.00	1.1320
102	Knoxville	Bull Run	Coal	760.00	0.9825
103	Bowling Gree	Paradise	Coal	858.00	1.0557
104	Bowling Gree	Paradise	Combined	364.17	0.4016
105	Bowling Gree	Paradise	Combined	364.17	0.3914
106	Bowling Gree	Paradise	Combined	364.17	0.4004
107	Mayfield	Shawnee	Coal	116.00	1.1617
108	Mayfield	Shawnee	Coal	116.00	1.1598

Appendix Table 2: Generator characteristics.

#	Location	Plant Name	Type	Max Capacity (MW)	CO2 Emissions (Tons/MWh)
109	Mayfield	Shawnee	Coal	116.00	1.1588
110	Mayfield	Shawnee	Coal	116.00	1.1541
111	Mayfield	Shawnee	Coal	116.00	1.1575
112	Mayfield	Shawnee	Coal	116.00	1.1389
113	Mayfield	Shawnee	Coal	116.00	1.1397
114	Mayfield	Shawnee	Coal	116.00	1.1397
115	Mayfield	Shawnee	Coal	116.00	1.1351
116	Starkville	Ackerman	Combined	348.92	0.4227
117	Starkville	Ackerman	Combined	348.92	0.4264
118	Starkville	Caledonia	Combined	263.67	0.4029
119	Starkville	Caledonia	Combined	283.17	0.4105
120	Starkville	Caledonia	Combined	267.17	0.4020
121	Johnson City	John Sevier	Combined	284.17	0.4135
122	Johnson City	John Sevier	Combined	284.17	0.4138
123	Johnson City	John Sevier	Combined	284.17	0.4117
124	Tupelo	Magnolia	Combined	318.00	0.4374
125	Tupelo	Magnolia	Combined	318.00	0.4382
126	Tupelo	Magnolia	Combined	318.00	0.4406
127	Memphis	South Haven	Combined	270.33	0.4074
128	Memphis	South Haven	Combined	285.92	0.4076
129	Memphis	South Haven	Combined	285.92	0.4039
130	Starkville	Kemper County	Natural Gas	83.00	0.7323
131	Starkville	Kemper County	Natural Gas	83.00	0.7163
132	Starkville	Kemper County	Natural Gas	83.00	0.7166
133	Starkville	Kemper County	Natural Gas	83.00	0.7110
134	Mayfield	Marshall Energy	Natural Gas	84.67	0.7350
135	Mayfield	Marshall Energy	Natural Gas	84.67	0.7224
136	Mayfield	Marshall Energy	Natural Gas	84.67	0.7033
137	Mayfield	Marshall Energy	Natural Gas	84.67	0.7200

Appendix Table 2: Generator characteristics.

#	Location	Plant Name	Type	Max Capacity (MW)	CO2 Emissions (Tons/MWh)
138	Mayfield	Marshall Energy	Natural Gas	84.67	0.7022
139	Mayfield	Marshall Energy	Natural Gas	84.67	0.7090
140	Mayfield	Marshall Energy	Natural Gas	84.67	0.7178
141	Mayfield	Marshall Energy	Natural Gas	84.67	0.7174

2.2 Wholesale electricity prices

The dispatch (supply) curve is built from the bottom up by aggregating the marginal cost curves for 141 generating units on the TVA grid. To find the parameters of the marginal cost function in equation 5, we estimate the cost function by fuel type and prime mover (generating unit) using the following equation:

$$TVC_{uv,\tau} = \alpha_1 MW_{uv,\tau} + \alpha_2 MW^2_{uv,\tau} + \epsilon_{uv,\tau} \tag{18}$$

where $TC_{uv,\tau}$ and $MW_{uv,\tau}$ represent the total cost ($/hour) and aggregate output (net generation in megawatts) for TVA plants with primary fuel type u and prime mover type v in month τ.

Appendix Table 3 shows the estimated coefficients for the cost function for each prime mover and fuel type combination. We set $a = \alpha_1$ and $b = \alpha_2$. The ACE model ”stacks” these linear marginal cost functions to create a piece-wise linear dispatch or supply curve. This piece-wise linear supply curve demonstrates increasing marginal costs which is a common characteristic of dispatch curves as more costly ”peaking” units are temporarily needed during periods of high demand. We also retire coal-fired generators in our simulations to reflect TVA's recent shift away from coal-fired power. According to TVA's announced retirement dates, we retired 760 MW in 2023, 1,701 MW in 2028, and 1,218 MW in 2030. These retirements change the curvature of the dispatch curve at discrete points in time.

Wholesale prices, w, are equivalent to the wholesale utility's average variable cost of electricity production. We input annual electricity production in MWh into equation 18 for each generating unit to calculate total annual variable costs, and sum each unit's costs to calculate total variable costs for the entire wholesale utility. We then divide annual total variable costs by the wholesale utility's total annual production in MWh to calculate the initial (time 0) annual average variable cost, w. The initial w is calculated directly from historic data. Wholesale costs are then updated after each annual model run based on simulated production

Appendix Table 3: Results of cost function estimate by fuel type and price mover

Fuel Type	Prime Mover	α_1($/MWh)	α_2($/MWhˆ2)
Bituminous	Steam turbine	12.92	0.001
Sub-bituminous	Steam turbine	13.710	0.001
Natural gas	Combustion turbine	29.766	0.001
Natural gas	Combined cycle	14.906	5.97E-8
Nuclear	Steam turbine	3.754	9.6E-5
Diesel	Combustion turbine	184.715	0.033
Diesel	Steam turbine	125.229	0.174

from each generating unit. w is therefore updated over time in the model as consumers adopt rooftop solar and the wholesale utility's generation mix changes.

2.3 Short-run electricity demand

To calculate the electricity demand at each retail utility, $q_j(H)$, we first gather hourly electric load data for the entire TVA region from FERC form 714. Form 714 data covers every hour from every year back to 2006. We use data from 2019 to construct representative hourly electricity demand for each retail utility in the region by dividing TVA load to each retail utility proportionally by population.

Hourly electricity demand is extremely inelastic; consumers do not alter their electricity usage based on the marginal cost of electricity generation, and retail utilities typically do not alter per-kwh electric rates based on the marginal cost of generation. We model inelastic hourly electricity demand by assuming a fixed quantity demanded, meaning the hourly demand curve is vertical for each hour of each day. We construct hourly demand in each retail utility from FERC 714 using the following formula:

$$q_j(H) = \frac{population_j}{\sum_k^J population_k} Load_{TVA} \tag{19}$$

where $Load_{TVA}$ is the total hourly load for the TVA region from FERC form 714. We subdivide hourly demand further into LMI and high-income hourly electricity demand by multiplying the hourly load in each retail utility by the proportion of retail utility consumers in each income category.

Appendix Figure 1 plots the quantity demanded for high-income and LMI residential customers in each retail utility. The demand curves represent the total amount of electricity demanded by each customer type. The curves demonstrate a familiar shape with lower demand during the night and higher demand during mid-day and evening. As expected, the two largest cities (Memphis and Nashville) consistently have the highest demands for each customer agent type. However, the retail customers differ in relative terms with Memphis demonstrating higher demand among low income and industrial customers while the more affluent,

service oriented Nashville shows higher demand among high income customers. The next three largest consumers (Knoxville, Huntsville, and Chattanooga) show similar trends in income-differentiated demand with Knoxville having the third highest demand from LMI residential customers but Huntsville having the third highest demand from high income customers.

We divide these total demands by the number of customers, I_{jz}, to obtain the average per capita demand $D_{jz}(H)$. We assume this average per capita demand is unchanged throughout our simulation so that total demand declines as I_{jz} declines.

2.4 Long-run electricity demand and retail electric rates

To initialize retail prices in the Ramsey pricing algorithm, we gather data from electricity sales for end-use regional electric utilities from EIA 861 Annual Electric Power Industry Reports. Form 861 data includes customer counts for residential, commercial, and industrial electricity consumers, along with electricity usage and revenues generated from each consumer group.

While the electricity market simulations assume hour-to-hour electricity demand is fixed, long-run year-to-year electricity demand will respond to changes in the retail price of electricity. This is consistent with evidence from our choice experiment which implies consumer demand for rooftop solar panels is responsive to the price of electricity. It is reasonable that other decisions, such as the decision to purchase energy-efficient appliances or energy efficient home improvements, are also responsive to the price of electricity in the long-run. Long-run demand for electricity should therefore be more price responsive than hourly demand. We estimate the elasticity of demand for each consumer group in each retail utility using a three-stage least squares log-log statistical model. This model corrects for simultaneity bias using an instrumental variables strategy to identify elasticities in group-level demand equations. Instruments for retail electricity prices include fuel costs for coal and natural gas, which shift wholesale electricity costs but are set on regional/global markets and plausibly exogenous from local electricity demand conditions. The last-stage estimated demand equations are:

$$log(q_{jz}) = c_{jz} - d_{jz} log(p_{jz}) \tag{20}$$

elasticity of demand estimates from this method average -.98 for residential consumers, -.97 for commercial consumers, and -1.26 for industrial consumers. These elasticities are consistent with outside literature such as Burke & Abayasekara (2018), which estimates a long-run elasticity of demand of approximately -1 for consumers nationwide, with the highest elasticities coming from industrial consumers. Other studies have

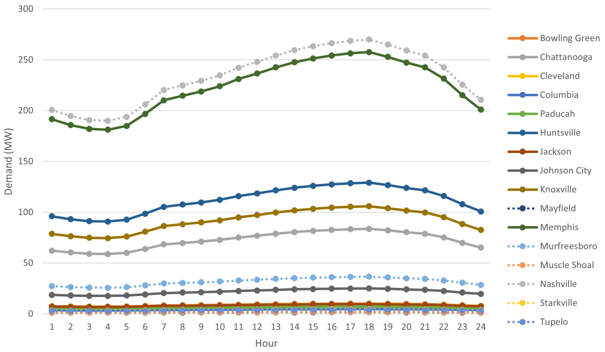

(a) High-Income

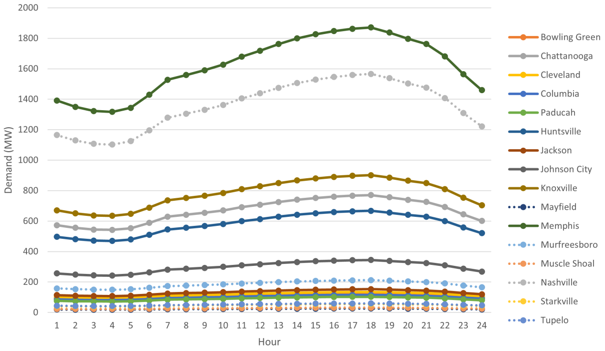

(b) Low- and Middle-Income

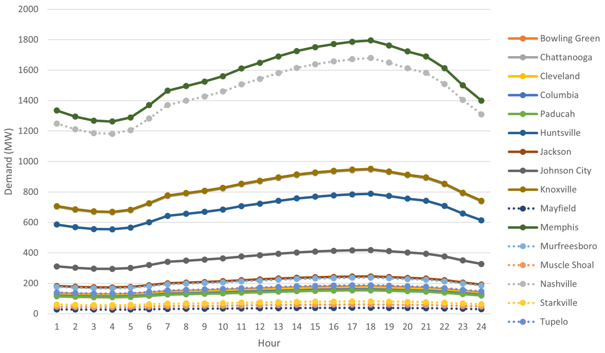

(c) Commercial

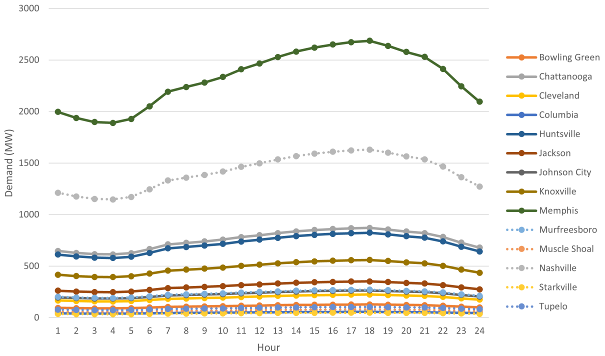

(d) Industrial

Appendix Figure 1: Retail utility hourly demand by customer agent type

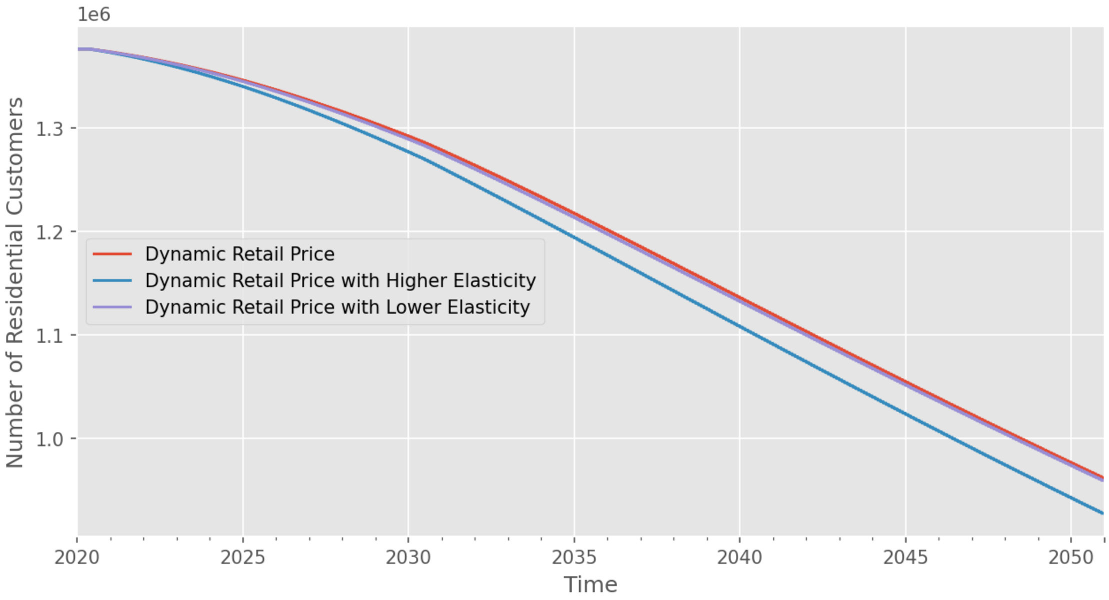

Appendix Figure 2: Sensitivity of PV adoption to 50% increase and decrease in electricity demand elasticity

Appendix Table 4: Sensitivity of residential TVA customer solar adoption to demand elasticity, 2021-2051

	Benchmark	50% increase	50% decrease
High income	34.88%	37.36%	35.11%
LMI	29.48%	31.99%	29.67%

shown long-run demand to be less elastic. A 2004 Meta-Analysis by Espey & Espey shows a median long-run elasticity estimate of -.81, with widely ranging estimates from -.04 to -2.25.

Notably, our estimated elasticities recover prices similar to those seen in actual TVA retail utilities during the time frame in which the model is calibrated, leading us to believe that the estimated elasticities are plausible. To examine the robustness of our estimates, we also simulate models in which the elasticity estimates (the d_{jz}'s) are doubled and halved for each consumer group in each retail utility. The initial retail price outputs our model generated using larger and smaller elasticities are much higher than real-world prices, unlike the price estimates generated from our benchmark model. The effects of different demand elasticities on solar adoption and the solar adoption gap between high-income and LMI customers are nonetheless small, as shown in Appendix Figure 2 and Appendix Table 4.

We construct the retail price of electricity, p_{jz} by dividing the annual revenue from electricity sales to each consumer group by the annual quantity of electricity sales to that group. This approach implicitly assumes that each consumer group pays a flat volumetric rate for electricity. Some retail utilities employ block pricing and tiered volumetric rates which increase with electricity use. However, Ito (2014) shows

that electricity consumers tend to respond to average rather than marginal electric rates, so assuming a flat electricity price equal to average prices is consistent with past evidence of how electricity consumption responds to electricity prices. The elasticity of demand for each group, $\epsilon_{jz} = -d_{jz}$, is easily calculated from the regression specification in equation 20 and then inserted into the pricing equation 14.

To illustrate how solar adoption impacts long-run demand, let $\bar{q}_{jz}$ be the average demand for an individual consumer in group z and retail utility j. We can rewrite equation 20 in terms of average demand and the number of consumers within the group who have not adopted rooftop solar, I_{jz}. Rewriting the demand function gives $log(I_{jz} * \bar{q}_{jz}) = c_{jz} - d_{jz}log(p_{jz})$, so $log(\bar{q}_{jz}) = c_{jz} - log(I_{jz}) - d_{jz}log(p_{jz})$. The elasticity of demand for an average individual consumer remains unchanged: $\frac{\partial \bar{Q}_{jz}}{\partial p_{jz}} \frac{p_{jz}}{Q_{jz}} = -d$. The elasticity of demand for a typical consumer will therefore not change as consumers adopt rooftop solar and I_{jz} decreases, so the elasticity of demand for each consumer group will not need to be updated within the agent based model as the number of consumers without rooftop solar decreases.

Finally, we calculate the fixed costs f_j for each local utility directly from EIA 861 data. Recall that we assume each utility is restricted to earning zero profits, which is equivalent to assuming that Revenue - Variable Cost - Fixed Cost = 0 for each utility, or that Fixed Cost = Revenue - Variable Cost: $f_j = \sum_z^Z p_{jz} * Q_{jz} - w \sum_{z=1}^Z Q_{jz}$. We can calculate f_j for each retail utility using total annual revenue for each consumer group and total annual electricity consumption for each consumer group reported directly in EIA 861 and our estimate for w using the process described above. Note that since the wholesale utility passes its own fixed costs (F) through to retail utilities in the form of a lump sum charge, the wholesale utility's fixed costs are incorporated directly into each retail utility's fixed cost, f_j. We do not need to know exactly how F is distributed across each retail utility, but instead only need to assume that pass-through of F to each retail utility does not change over time.

2.5 Fixed Cost Validation

Using fixed costs implied by EIA electricity sales revenue data and an annual zero-profit condition is consistent with our pricing model, and offers us a method to estimate retail utility-level fixed costs for which there is no available direct data. However, we also make an effort to ensure that these fixed costs represent a reasonable approximation of actual fixed costs faced by retail utilities.

The fixed costs passed on to consumers through retail electric rates include both fixed costs incurred by the wholesale utility and passed onto retail utilities, including investment and maintenance costs for generation resources and transmission lines, and fixed costs incurred at the retail utility level, including

Appendix Table 5: Implied retail utility Fixed Costs

retail utility	Implied Fixed Cost	$ Fixed Cost/mwh
Bowling Green	64,635,569	75.59
Chattanooga	413,957,450	67.17
Cleveland	76,601,463	71.40
Columbia	47,610,961	77.42
Paducah	44,719,712	77.42
Huntsville	331,917,813	63.86
Jackson	114,104,458	69.99
Johnson City	142,480,206	72.92
Knoxville	404,975,015	67.24
Mayfield	10,910,862	91.53
Memphis	1,045,396,861	72.99
Murfreesboro	103,357,897	70.94
Muscle Shoal	19,903,882	76.97
Nashville	918,069,135	70.29
Starkville	28,704,243	71.68
Tupelo	48,643,220	69.40

maintaining distribution lines and metering equipment. While we have no data on fixed costs at the retail utility-level for the locations in our model, we do have some data on fixed costs from the TVA, which we supplement with data from FERC form 1 from investor-owned utilities (IOUs) in locations outside the scope of our model. We then compare TVA data and FERC data from investor-owned utilities to create a benchmark for the total fixed costs faced by an LPC, which we compare to the implied fixed costs used in our model.

According to the TVAs 2023 cost of service study, total fixed (non-energy) costs amounted to $8.3 billion, or approximately $53/mwh. We use this as a benchmark approximation of TVA-level per-mwh fixed costs.

For retail utility-specific costs, we turn to FERC form 1 data on IOUs. These data include categories for annual operations and maintenance costs, and suggest that the median annual fixed cost for an IOU in 2019 (the most recent year in the data) is approximately $22/mwh. Unfortunately, none of the retail utilities in our model are included in the FERC data set, since none are investor-owned. However, if retail utilities have similar cost structures to IOUs, then the FERC data still provides a useful benchmark for retail fixed costs.

According to the combined TVA cost of service study and the FERC IOU data, a reasonable benchmark for the total fixed costs passed onto retail customers is $53/mwh + $22/mwh = $75/mwh, which we compare to the implied $/mwh fixed costs used in our model. Appendix Table 5 shows the implied fixed costs on a per mwh basis used in our simulation.

The mean implied fixed cost is $73.19/mwh, while the median is $71.54. Both are close to the $75 benchmark fixed cost from the TVA and FERC data. In addition, no retail utility in our model has an

implied fixed cost lower than the $53 fixed cost passed on by the TVA, meaning no retail utility has an unreasonably low implied fixed cost estimate, nor do any retail utilities have extremely high implied fixed costs. For these reasons, we argue that the implied fixed costs in our model are a plausible approximation of actual retail fixed costs.

2.6 Choice experiment and solar adoption

We conducted a survey of $2,307$ households in the TVA region. The survey consisted of around 40 questions and took respondents about fifteen minutes to complete. The survey included a stated-preference experiment, which presented six choice experiments each comparing three hypothetical solar arrays that vary in three dimensions: cost, electricity savings, and carbon emissions-reductions. After the stated preference experiment, respondents were presented a battery of questions about their opinions and experience with roof top solar panels. Finally, respondents answered a handful of socio-demographic questions.

Our sample frame is the TVA region, incorporating all of Tennessee and parts of six neighboring states. Respondents were collected through the Qualtrics panel which provides a channel to distribute surveys. We asked Qualtrics to sample at the zip code level, so that the number of respondents in each zip code roughly matches the distribution of households across the TVA region. Qualtrics markets the survey to its standing panel of respondents and pays a small fee for each completed survey. In areas where initial response is below the proportion needed they conduct additional marketing and sometimes increase the fee they pay to respondents.

Each respondent was presented with some general information about solar panel arrays and the solar panel characteristics used in the experiment. Then they were given a set of six consecutive hypothetical choices, each consisting of three potential solar arrays and asked which one they were most likely to purchase. The solar arrays were characterized by three attributes: *payment*, *savings*, and *abatement*. The *payment* variable was the amortized monthly cost of the installed solar array over twenty-five years. The *savings* variable, measured in dollars, represented the monthly savings from utility rates avoided after solar adoption over the next twenty five years. The savings minus payment was also presented separately with colored font, red if the cost exceeds the savings and green if the savings were greater than the cost. Respondents also saw a randomly generated reduction in emissions, *abatement* reported in percentages. An example choice experiment question is presented in Appendix Figure 3.

After making their choice, respondents were asked how likely they were to actually purchase their most preferred array along with follow up questions about when they would purchase it and how uptake in their

Characteristics	Option 1	Option 2	Option 3
**Payment for Solar Option**	$80/mo for 25 yrs	$31/mo for 25 yrs	$20/mo for 25 yrs
**Savings on Electric Bill**	$16/mo for 25 yrs	$43/mo for 25 yrs	$20/mo for 25 yrs
**Savings Minus Payment**	$-19200 over 25 yrs	$+3600 over 25 yrs	$+0 over 25 yrs
**Reduction in Emissions**	54%	51%	43%

Appendix Figure 3: Example Choice Experiment Solar Array Characteristics. Respondents were presented with six different choice experiments. The solar array characteristics were presented in this way each time. The values of each of the payment, savings and abatement were randomly generated. Savings minus payment was calculated from those two numbers and color coded where green indicated net savings and red indicated net payments. All random numbers were drawn from independent uniform distributions.

community would affect their decision to purchase. Afterwards, they were presented with the next set of three arrays.

Each of the three solar array characteristics was randomly drawn from an independent uniform distribution. Monthly system cost varied between $10 and $90, monthly electricity savings varied between $0 and $150, and emissions-reductions varied between $0 - 100\%$. Those ranges are somewhat beyond the typical cost and electricity savings produced by solar panels installed in the region, but we wanted to ensure that we had reasonable data coverage across the entire state space. Similarly, we chose to use independent draws from the three distributions meaning that some systems appeared with very high cost and low savings or high emissions-reductions and low electricity cost savings. These particular scenarios are unlikely and in some cases clearly dominated by other choices. We decided those odd solar arrays were a worthwhile sacrifice to ensure we had independent variation in all three dimensions. This variation provides the ability to separately identify the way that cost, savings and emissions-reductions affect the decision to purchase solar. The attributes and their domains are summarized in Appendix Table 6 below.

We created survey responses for each retail utility by linking respondent zip codes to the TVA Customer Service Districts. We further disaggregated the sample by using respondent self-reported incomes to create

Appendix Table 6: Solar Panel Attribute Characteristics

Attribute	Description	Domain
Payment	Choosing a solar option results in an additional monthly cost on top of your current electric bill.	\$10 to \$90
Savings	Choosing a solar option results in savings on your electric bill, which is expressed as a monthly average. The savings will occur over the average lifetime of a rooftop solar system (about 25 years).	\$0 to \$150
Savings Minus Payment	Net savings are expressed over the lifetime of the system or contract. When savings are greater than the payment, the difference is positive shown in green with a (+) sign. When savings are less than payments, the difference is negative shown in red with a minus (–) sign.	-\$90 to \$140
Emissions-Reductions	The percent reduction of emissions from your use of electricity.	0% to 100%

Appendix Table 7: Summary statistics for choice experiment sample (2,307 respondents)

Variable	Median
Age	42-45
Female	71%
Income	\$35,000-\$49,999
Own Home	55%
Square Footage	1,000-1,999
Know Someone with Panels	19%
Have (or Had) panels	1%

a high-income ($> \$150,000$ annual salary) and low-to-moderate income (LMI) sample in each retail utility. We then estimated the following model for the probability of solar adoption for each of our retailer-income subsamples

$$\begin{aligned} Pr[adopt]_{jz} = \beta_{0;jz} + \beta_{1;jz} payment_{jz} + \beta_{2;jz} savings_{jz} + \beta_{3;jz} abatement_{jz} + \beta_{4;jz} age_{jz} \\ + \beta_{5;jz} gender_{jz} + \beta_{6;jz} education_{jz} + \beta_{7;j} solar_{jz} + \beta_{8;jz} housing_{jz} + \epsilon_{jz} \end{aligned} \tag{21}$$

where age_{jz}, $gender_{jz}$, and $education_{jz}$ are the demographics of LMI and high-income respondents in retail utility j, $solar_{jz}$ is a vector of variables measuring a respondent's experience and familiarity with rooftop solar, and $housing_{jz}$ is a vector of variables representing the respondent's home characteristics and ownership status.

We use McFadden's (1973) conditional logit (CL) model to estimate coefficient estimates, β_{jz} for each retail utility and each customer type. We then use these estimated models to parameterize a solar adoption behavioral rules in equation 2 that vary across retail utilities and customer agent types. Specifically, we set $\pi_{1,jz} = \beta_{1;jz}$, $\pi_{2,jz} = \beta_{2;jz}$, $\pi_{3,jz} = \beta_{3;jz}$ and

$$\pi_{0,jz} = \beta_{0;jz} + \beta_{4;jz} \overline{age}_{jz} + \beta_{5;jz} \overline{gender}_{jz} + \beta_{6;jz} \overline{education}_{jz} + \beta_{7;j} \overline{solar}_{jz} + \beta_{8;jz} \overline{housing}_{jz} \tag{22}$$

where lines over a variable indicate the variable is evaluated at the retailer-income subsample mean. Estimated values for the weights for the solar adoption behavior rules are shown in Appendix Tables 8-10. We assume the commercial agent adopts according to the average residential agent and use the pooled residential agent sample to estimate adoption coefficients for the commercial agent. Because industrial agent adoption is likely driven by considerations that differ from residential agents, an industrial agent adoption rule based on a survey of residents is unlikely to accurate reflect industrial solar adoption behaviors. A similar survey of industrial customers could be used to specify a solar adoption rule for this customer agent type. Absent these survey results, we assume no industrial solar adoption.

Consistent with our hypotheses we find $\pi_{1,jz} < 0$, $\pi_{2,jz} > 0$, and $\pi_{3,jz} > 0$ for all customer types in each retail utility. Consistent with previous choice experiment research (Dong and Sigrin, 2019; Petrovich et al., 2021), we also find that adoption is more sensitive to upfront PV system costs than to future electricity bill savings.

The customer agent's probability of solar adoption and the number of customers adopting respond to the utility agent's generation decision which impacts the pollution intensity of the grid and the retail utility agent's choice for the retail electricity rate. For instance, $savings_{jz,t} = p_{jz,t} * S_{jz}$ updates as retail utility

Appendix Table 8: High-Income Residential Adoption Coefficients

Name	Intercept $\pi_{0,jz}$	Cost Coefficient $\pi_{1,jz}$	Savings Coefficient $\pi_{2,jz}$	Abatement Coefficient $\pi_{3,jz}$
Bowling Green	0.3976	-0.0263	0.0135	0.0115
Chattanooga	-0.0229	-0.03	0.0169	0.0122
Cleveland	13.4756	-0.0316	0.0148	0.0167
Columbia	0.8882	-0.0272	0.0144	0.0122
Paducah	-0.5093	-0.0231	0.0183	0.0046
Huntsville	-0.1448	-0.0255	0.0123	0.0071
Jackson	-0.8993	-0.0268	0.0149	0.0046
Johnson City	13.4245	-0.0349	0.015	0.0094
Knoxville	-0.5881	-0.0291	0.0142	0.0125
Mayfield	0.2949	-0.0361	0.0108	0.0223
Memphis	0.3239	-0.0223	0.0115	0.0089
Murfreesboro	-0.5966	-0.0295	0.0132	0.0077
Muscle Shoal	15.1996	-0.0372	0.0184	0.0082
Nashville	-0.1879	-0.0254	0.013	0.0107
Starkville	-0.0795	-0.0311	0.0133	0.0089
Tupelo	10.8647	-0.0193	0.012	0.0044

Appendix Table 9: LMI Residential Adoption Coefficients

Name	Intercept $\pi_{0,jz}$	Cost Coefficient $\pi_{1,jz}$	Savings Coefficient $\pi_{2,jz}$	Abatement Coefficient $\pi_{3,jz}$
Bowling Green	0.1452	-0.0263	0.0135	0.0115
Chattanooga	-0.3337	-0.03	0.0169	0.0122
Cleveland	-0.3445	-0.0316	0.0148	0.0167
Columbia	-0.4548	-0.0272	0.0144	0.0122
Paducah	-0.5093	-0.0231	0.0183	0.0046
Huntsville	0.1229	-0.0255	0.0123	0.0071
Jackson	-0.0864	-0.0268	0.0149	0.0046
Johnson City	-0.3219	-0.0349	0.015	0.0094
Knoxville	-0.6015	-0.0291	0.0142	0.0125
Mayfield	0.2949	-0.0361	0.0108	0.0223
Memphis	-0.0241	-0.0223	0.0115	0.0089
Murfreesboro	-0.221	-0.0295	0.0132	0.0077
Muscle Shoal	-1.5335	-0.0372	0.0184	0.0082
Nashville	-0.3983	-0.0254	0.013	0.0107
Starkville	0.412	-0.0311	0.0133	0.0089
Tupelo	-0.1204	-0.0193	0.012	0.0044

Appendix Table 10: Commercial and Industrial Adoption Coefficients

Name	Intercept $\pi_{0,jz}$	Cost Coefficient $\pi_{1,jz}$	Savings Coefficient $\pi_{2,jz}$	Abatement Coefficient $\pi_{3,jz}$
Bowling Green	-1.3545	-0.0211	0.0113	0.0116
Chattanooga	-1.1488	-0.0247	0.0129	0.0085
Cleveland	-1.2239	-0.0272	0.0122	0.0125
Columbia	-1.077	-0.022	0.01	0.0089
Paducah	-1.0941	-0.0215	0.0143	0.0017
Huntsville	-1.1348	-0.0189	0.0104	0.0069
Jackson	-0.9939	-0.0212	0.0112	0.0038
Johnson City	-0.8194	-0.0257	0.0111	0.0041
Knoxville	-0.8491	-0.024	0.0094	0.006
Mayfield	-1.4868	-0.0317	0.0102	0.0237
Memphis	-1.1686	-0.018	0.0098	0.0076
Murfreesboro	-0.7904	-0.0229	0.009	0.005
Muscle Shoal	-0.3746	-0.0284	0.0089	-0.0021
Nashville	-0.9648	-0.0218	0.0098	0.0067
Starkville	-1.1562	-0.0271	0.0117	0.0103
Tupelo	-1.1215	-0.0195	0.0108	0.0073

agents set new retail prices $p_{jz,t}$ with the quantity of electricity generated from a solar system, S_{jz}, based on PVwattts estimates in each retail utility. The variable $abatement_{jz,t}$ is calculated as

$$abatement_{jz,t} = e_t \frac{Z_{jz,t} - S_{jz}}{Z_{jz,t}} \tag{23}$$

where $e_t = \frac{\sum_{k=1} E_{k,t}}{\sum_{k=1} q_{k,t}}$ is the average emission intensity of electricity generation, $q_{k,t}$ is the amount of electricity TVA generated at node k and $E_{k,t}$ is the pollution emissions generated at node k. $Z_{jz,t} = Q_{jz,t}/I_{jz,t}$ is the average customer usage of electricity in retail utility j, and $Q_{jz,t}$ is the amount of electricity consumed by customer type z in retail utility j.

In line with the structure of the choice experiment, the variable $cost_t$ is the monthly payment for a loan to finance the total cost, TC, of a PV+battery system over 25 years with a 6% interest rate (Feldman and Schwabe, 2018). The total cost of a PV+battery system is calculated as

$$\text{TC}_t = \text{Size}_{PV}(W) \times \text{Cost}_{t,PV}\left(\frac{\$}{W}\right) + 2.5 \times \text{Size}_{\text{Batteries}}(Wh) \times \text{Cost}_{t,\text{batteries}}\left(\frac{\$}{Wh}\right) \tag{24}$$

The multipliers for the cost are considering PV panels with a lifetime of 25 years and batteries with lifetime of 10 years (Haruna et al., 2011). Accordingly, to maintain a PV system for 25 years, the batteries would need to be replaced 2.5 times.

The size of the PV panels and battery necessary to defect from the grid are calculated as

$$\text{Size}_{PV} = \frac{\text{Daily Demand}}{4\left(\frac{\text{Peak Sun Hours}}{\text{Day}}\right) \times 86\%} \tag{25}$$

$$\text{Size}_{\text{Batteries}} = \frac{\text{Daily Demand}}{75\%} \tag{26}$$

The required size of the PV panels, in watts, is a function of the needed daily demand, number of peak sun hours, and efficiency. Most states in the US receive an average of 4 sun hours. The efficiency parameter reflects losses due to system degradation, dust, weather, etc, which are assumed to be 14% (NREL, 2021). The lithium ion battery size is suitable to supply electricity for an entire day with an efficiency of 75% (Omar et al., 2015; Tervo et al., 2018) The per unit cost of PV panels and batteries are falling over time to match the significant cost decreases in previous decades. This cost decrease was achieved by fitting the following equation

$$\text{Cost}_t = e^{a+bt} + c \tag{27}$$

to historic prices of PV panels (Barbose at al., 2019) and lithium ion battery prices (Bloomberg NEF, 2019). The fitted curves and actual data are shown in Appendix Figures 4 and 5, for PV panels and batteries, respectively. The R^2 scores of the fits are 0.86 and 0.98.

Assuming customers are more likely to adopt solar at the same time they replace their roof, we assume customers update their solar adoption decision every 20 years which is approximately the lifetime of asphalt roof shingles.

2.7 Transmission line calibration

An exhaustive search was performed using a discretized line reactance variable

$$Reactance_{km} \in [0.1, 10, 20, 30, 40, 50, 60, 70, 80, 90, 100] \tag{28}$$

and a factor to increase the capacity of each line

$$Capacity_{km} = Factor_{km} \times Capacity_{km,original} \tag{29}$$

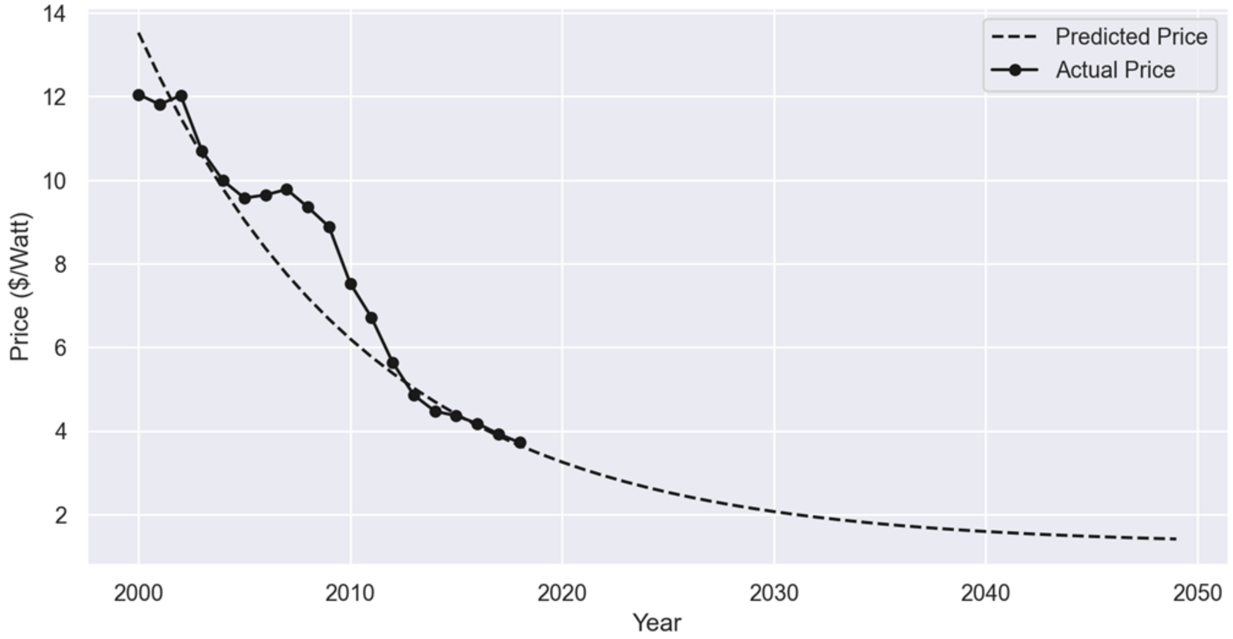

Appendix Figure 4: Curve fitting for PV panel price. Actual price from

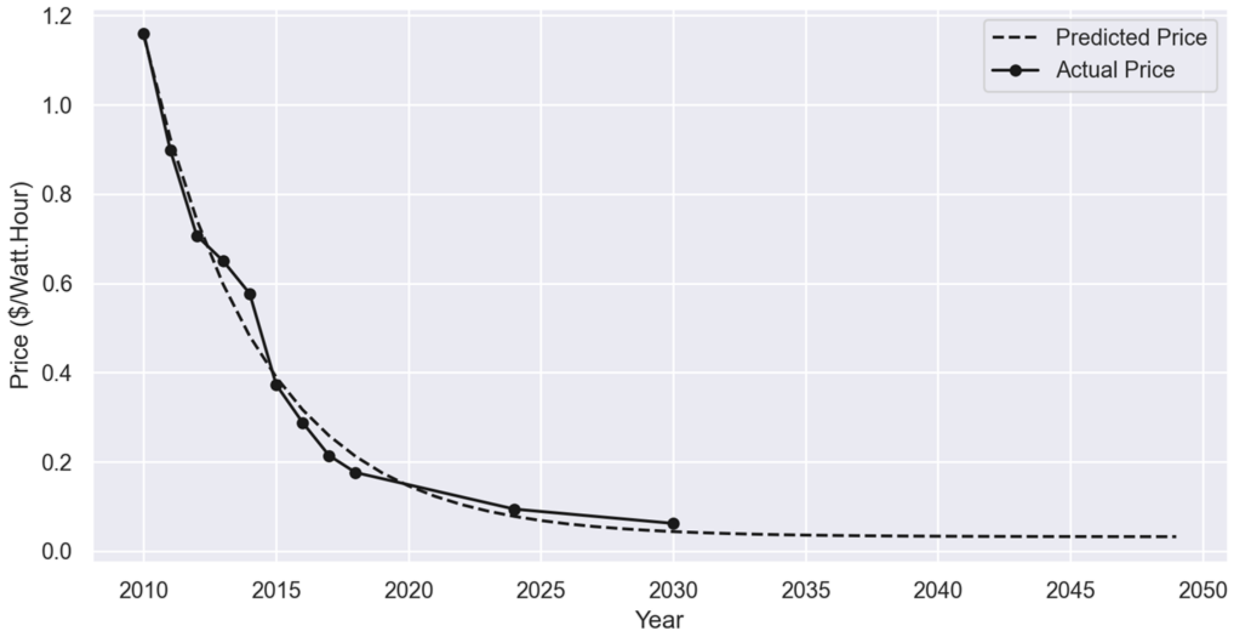

Appendix Figure 5: Curve fitting for battery price.

Appendix Table 11: Line Capacities and Reactances after Calibration

Line Number	from	to	Cap	Reactance
Line 1	1	9	831.676	60
Line 2	1	10	2681.4504	0.1
Line 3	1	12	3505.9664	20
Line 4	1	14	6201.677	60
Line 5	2	3	11389.67	30
Line 6	2	6	15162.998	60
Line 7	2	12	6255.196	90
Line 8	3	9	24619.04	0.1
Line 9	3	12	5181.933	60
Line 10	4	7	19308.423	70
Line 11	4	12	29954.525	40
Line 12	4	13	3229.529	0.1
Line 13	4	14	9155.528	70
Line 14	4	15	1230.0465	70
Line 15	5	10	667.4493	50
Line 16	6	12	944.2557	20
Line 17	6	13	9610.23	90
Line 18	7	10	1046.932	40
Line 19	7	11	33518.315	50
Line 20	7	13	440.5436	50
Line 21	7	16	2641.5048	20
Line 22	8	9	3791.008	30
Line 23	9	12	5594.982	70
Line 24	10	14	6734.265	30
Line 25	11	16	6169.218	90
Line 26	12	14	35344.504	0.1
Line 27	13	15	4597.804	100
Line 28	13	16	8656.338	0.1
Line 29	15	16	6458.367	100

where $Factor_{km} \in [1, 2, 3, 4, 5]$. This is necessary because the agglomeration of buses requires a large increase in transmission lines capacities to withstand the new power flow. Finally, the solution from the DC-OPF solver with the lowest mean squared error between the calculated generator plants commitments and the actual commitments from historical data is selected. The final line capacities and reactances are shown in Appendix Table 11 where "from" and "to" refer to the node numbers.

3 Benchmark Model Validation

Model validation is a process that determines whether the model output is a reasonably accurate representation of the real world. We have taken several steps to conduct predictive validation of the individual agent behaviors produced from our model. First, we conducted several focus groups with TVA personnel to compare our model's system-level output with domain experts. TVA personnel were selected from two

categories to encompass the supply and demand sides of TVA's grid. First, were personnel with experience in resource planning that gives them a big picture overview of the historic and potential future operation of the TVA grid. Second, were TVA personnel that work closely with local power companies and end-use customers. We presented model results in both animation and graphical formats and asked TVA personnel to identify results that were consistent with their understanding of the TVA grid and those that were not. We also asked TVA personnel to list any data or forecasts they were implicitly using to assess our model results. This exercise allowed us to re-assess our modeling assumptions, validate our conceptual model, and identify previously overlooked datasets.

To validate individual agent behaviors, we engaged in a series of model-to-model comparisons. For the utility agent behavior, we compared hourly unit-level dispatch predictions from the OPF component of our agent-based model to hourly OPF output used by TVA for general operations in 2019. Comparisons were based on total daily generation as well as hourly minimum and hourly maximum generation for summer peaking peaking demand profile. Dispatch at each generating unit in the model was within 10% of the TVA OPF measures.

To validate residential agent solar adoption behavior, we compared benchmark model predictions of distributed solar adoption capacity to distributed solar adoption forecasts used by TVA in their most recent Integrated Resource Plan. The forecasting approach used by TVA is similar to the NREL Distributed Market Demand Model, which simulated potential adoption of a given resources as a function of payback period. More information on TVA's distributed solar adoption forecasts is available on TVA's Integrated Resource Planning webpage. To account for a variety of future uncertainties, TVA considers 6 scenarios each paired with 5 strategies for a total of 30 possible futures. Scenarios describe the potential futures TVA may find itself operating in while strategies depict business approaches TVA could employ to meet energy demand in these future worlds. The mean adoption in 2050 across all 30 combinations of scenarios and strategies is 3,354 MW. Given the median size of residential solar systems around the TVA service area is 8.5 kW (Barbose et al. 2023), our model predicts 3,509 MW of residential solar adoption in 2051. This forecast is within the upper and lower quartile (2,504-3,825 MW) of TVA's residential solar adoption forecasts. While accurate zip code level solar adoption data does not exist to validate our city-level adoption forecasts, this model-to-model comparison at the system level indicates that residential agent behaviors in our model are consistent with TVA's forecasts.

To validate retail utility behavior, we conduct historical data validation by comparing model predicted retail prices to EIA Electric Power Monthly data on residential retail electricity prices. 2020 residential retail prices in our simulation are 9.0 cents/kWh which is 18% higher than 2020 EIA data for the TVA service area.

2020 retail prices across all sectors (residential, commercial, industrial) in our simulation are 8.0 cents/kWh which is 16% higher than 2020 EIA data.

4 Choice experiment survey questionnaire

INTRODUCTION You are invited to participate in our study, which aims to understand residents' intention to adopt solar panel and electricity usage behavior in Tennessee. You can participate in this study even if you don't own a solar panel now. Our study is not connected with your local electricity company/provider so it won't affect your utility bills or any other decisions.

INFORMATION ABOUT PARTICIPANTS' INVOLVEMENT IN THE STUDY You will spend about 15 minutes to complete this online survey. First, we will ask your preferences on the several options regarding type of solar panel, cost, carbon emission, electricity saving and so on. Second, we will also ask your experiences with solar energy, attitudes toward solar energy, electricity habit, privacy concern and so on. Finally, we will ask demographic information.

RISKS The risks and discomfort associated with participation in this study are no greater than those ordinarily encountered in daily life or during any surveys. If you feel tired when answering the questions, you may stop at any point and come back later.

BENEFITS You may not directly benefit from your participation in this study. Your answers will help us understand the reasons of solar penal adoption. Your responses may help decision makers improve future solar energy adoption. PAYMENT You will receive compensation from your panel provider, in the form of reward points that are between the values of .50 - 2.

CONFIDENTIALITY No sensitive questions will be asked during the survey, and no personal identifiable information will be collected. In other words, NO participants in our survey can be identified by the answers to our questions. All collected data will be kept confidential, stored securely, and will be made available only to persons conducting the study.

CONTACT If you have questions at any time about the study or the procedures, you may contact the researcher, Charles Sims, at 508 Min H. Kao Building, 1520 Middle Dr., University of Tennessee, Knoxville, or (865) 974-3787. If you have questions about your rights as a participant, contact University of Tennessee, Knoxville IRB at (865) 974-7697, utkirb@utk.edu, or visit the office at 1534 White Avenue.

PARTICIPATION Your participation in this study is voluntary; you may decline to participate without penalty. If you decide to participate, you may withdraw from the study until you submit the survey without

penalty and without loss of benefits to which you are otherwise entitled. After you submit the survey, we cannot remove your responses because we will not know which responses came from you. Click on the "I agree" box at the bottom of the page constitutes your consent to participate. You may also click the "print" button to have a copy of this statement.

o I agree

o I do not agree

Q1. Are you a student?

Q2. What is your age?

Q3. What is your gender?

Q4. What is your household's annual income before tax on average?

Q5. What is your zip code that you are currently living in?

Q6. We care about the quality of our data. In order for us to get the most accurate measures of your opinions, it is important that you thoughtfully provide your best answers to each question in this survey.

o I will provide my best answers

o I will not provide my best answers

o I can't promise either way

We would like to introduce you to the concept of solar panels:

Solar panels use sunlight to make electricity. When sunlight shines on the solar panels they convert the sun's rays into electricity for your home. If your solar panels generate more electricity than you need, you can sell your extra electricity to your electricity provider or receive credit on your electricity bill.

You'll be asked a series of questions about your preferences for residential solar options with different characteristics. The remainder of this section describes these residential solar characteristics.

Residential Solar Characteristics In the following section, you'll be asked a series of questions about your preferences for residential solar options with different characteristics. The following describes these residential solar characteristics.

Payment for Solar Option The solar panel options described here are paid for on an installment plan as part of your electric bill. Choosing a solar panel installation option results in an additional monthly cost on top of your current electric bill. That cost covers the cost of the panels and their installation. After 25 years the system is paid off and there are no additional costs.

Savings on Electric Bill Choosing a solar option results in savings on your electric bill. We report those savings as a monthly average reduction in electricity spending. These savings will occur over the average lifetime of a rooftop solar system (about 25 years). The amount of savings depends on the amount of sunshine in the area, the solar panel's technology and the cost of electricity.

Savings Minus Payment Net savings are expressed over the lifetime of the solar panel system by comparing the monthly cost to the average monthly savings. If over the life of the plan savings are greater than the payment, the difference is positive shown in green with a (+) sign. When savings are less than payments, the difference is negative shown in red with a minus (–) sign.

Reduction in Emissions Electricity usage leads to pollution emissions including sulfur dioxide, particulate matter and greenhouse gasses. These pollutants can cause health problems, smog and are a contributing factor to climate change. Installing solar panels reduces the emissions of all of these pollutants. For each scenario we will report the percentage reduction in pollution from choosing to install solar panels.

These emissions savings are determined by how much power a solar system generates and the amount and type of fossil fuels used to generate electricity in your area. Installing a solar panel in a utility district that relies heavily on renewable energy will not reduce emissions very much. Installing a solar panel in a pollution intensive utility district will lead to larger reductions in emissions.

The following section will consist of six scenarios. Please select your preferred option in each scenario one. Each scenario is separate so do not let your answers on one scenario affect your response on the next ones.

Scenario 1 Please identify which option you would prefer and whether you would actually choose that option. Base your choice on the options on this page only.

Characteristics	Option 1	Option 2	Option 3
**Payment for Solar Option**	$80/mo for 25 yrs	$31/mo for 25 yrs	$20/mo for 25 yrs
**Savings on Electric Bill**	$16/mo for 25 yrs	$43/mo for 25 yrs	$20/mo for 25 yrs
**Savings Minus Payment**	$-19200 over 25 yrs	$+3600 over 25 yrs	$+0 over 25 yrs
**Reduction in Emissions**	54%	51%	43%

Q7. Which option would you prefer?

o Option 1

o Option 2

o Option 3

Q8. Would you actually choose the option selected above or not install solar panels?

Q9. Would you actually choose this option if XX% of your neighborhood have solar?

[PREVIOUS QUESTION REPEATED FIVE MORE TIMES WITH DIFFERENT NUMBERS FOR EACH OPTION]

Q24. Please tell us if you agree or disagree with the following statement:

I consider my household energy usage data as private.

Q25. Please tell us if you agree or disagree with the following statement: I am concerned that my utility company can infer when someone is at home and other lifestyle information from my household energy usage data.

Q26. Please tell us if you agree or disagree with the following statement: I consider the temperature setting data of my heating and cooling as private.

Q27. Please tell us if you agree or disagree with the following statement: I am concerned that my utility

company can infer when someone is at home and other lifestyle information from my heating and cooling's temperature setting data

Q28. Please tell us if you agree or disagree with the following statement: My utility company always protects customer's privacy.

Q29. Please tell us if you agree or disagree with the following statement: My utility company always keeps customers' best interests in mind.

Q30. Please tell us if you agree or disagree with the following statement: My utility company always keeps their promises.

Q31. If you were to install a solar panel, how likely would you be to allow your utility company to install a new electricity meter so that both you and your utility company know the source of your electricity consumption (i.e., from your solar panels vs. the power grid) at all times?

Q32. How satisfied are you with your current electric price?

Q33. How likely would you be to participate in a demand response program that encourages you to reduce energy usage during peak hours (e.g., 5-8pm) or shift energy usage from peak to off-peak hours in exchange for a money reward, e.g., 30? $Ingeneral, off-peakhourshavealowerelectricityrate.$

Q34. Please tell us the first three words or phrases that come to mind when you think about residential solar.

Q35. Which of the following statements best describes you when you first purchase a product that has new and/or advanced technologies (e.g., iPad, HD TV, electric vehicle, highly efficient appliances, etc.)?

Q36. Do you know anyone with solar panels on their home?

Q37. Have you ever had solar panels on your home? Why not? Check all the explanations that apply.

o Not enough sun light

o Don't like solar power

o Don't own home

o Too expensive

o Worried about roof damage

o Never considered solar panels

Q38. Do you own or rent your house/apartment?

Q39. How many more years do you expect to live at your current residence?

Q40. Which of the following best describes the type of home you live in? (Please select one category.)

Q41. In your household, which of the following activities are you involved in? (Please select all that apply.)

o Reviewing and/or paying the monthly electric bill

o Making decisions about programs, payments, and other options provided by your electricity provider

o Making decisions about solar panels at your house

o None of the above

Q42. How large is your home?

Q43. Please indicate the highest level of education you have completed or are in the process of completing.

Q44. Which is your ethnic background? Select all that apply.

Q45. What is your current employment status?

5 Additional Simulation Results

Appendix Table 12: Percent decrease in TVA residential customers between 2021-2051 due to solar adoption and amount of solar adoption (in percentage points) attributable to the utility death spiral and federal solar rebate

		Adoption	death spiral	federal rebate
TVA service area	High-income	34.88%	0.78	5.89
	LMI	29.48%	0.81	6.09
Tupelo, MS	High-income	79.39%	0.00	0.00
	LMI	37.67%	1.07	5.85
Starkville, MS	High-income	34.06%	0.13	9.69
	LMI	35.62%	0.67	8.61
Paducah, KY	High-income	29.03%	0.00	2.71
	LMI	32.46%	0.85	6.33
Nashville, TN	High-income	30.97%	0.64	5.95
	LMI	27.20%	0.66	5.59
Muscle Shoals, AL	High-income	64.60%	0.00	0.00
	LMI	8.84%	0.35	4.06
Murfreesboro, TN	High-income	18.43%	0.39	5.36
	LMI	23.86%	0.77	6.05
Memphis, TN	High-income	42.53%	0.67	6.54
	LMI	35.70%	0.72	6.29
Mayfield, KY	High-income	0.00%	0.00	0.00
	LMI	12.27%	0.05	4.56
Knoxville, TN	High-income	20.81%	1.04	5.38
	LMI	20.57%	0.72	5.12
Johnson City, TN	High-income	78.29%	0.00	0.00
	LMI	20.14%	0.65	6.18
Jackson, TN	High-income	19.39%	0.00	2.94
	LMI	34.27%	1.35	6.89
Huntsville, AL	High-income	32.14%	1.31	6.33
	LMI	37.27%	1.29	6.74
Columbia, TN	High-income	44.00%	0.00	4.25
	LMI	20.75%	0.86	5.48
Cleveland, TN	High-income	78.57%	0.00	0.00
	LMI	25.35%	0.82	6.72
Chattanooga, TN	High-income	34.18%	1.27	7.61
	LMI	28.32%	1.05	6.76
Bowling Green, KY	High-income	39.66%	0.00	3.60
	LMI	36.79	0.88	6.99

Appendix Table 13: Percent decrease in number of residential TVA customers due to rooftop solar adoption, 2021-2051

	Benchmark	Fixed prices	No rebate	Higher solar irradiance
All residential	30.13%	29.32%	24.06%	38.42%
High income	34.88%	34.10%	29.00%	42.63%
LMI	29.48%	28.67%	23.40%	37.85%

Notes: The utility death spiral is found by taking the difference between columns A and B. The effect of the federal solar subsidy is found by taking the difference between columns A and C. The effect of solar irradiance is found by taking the difference between columns A and D.

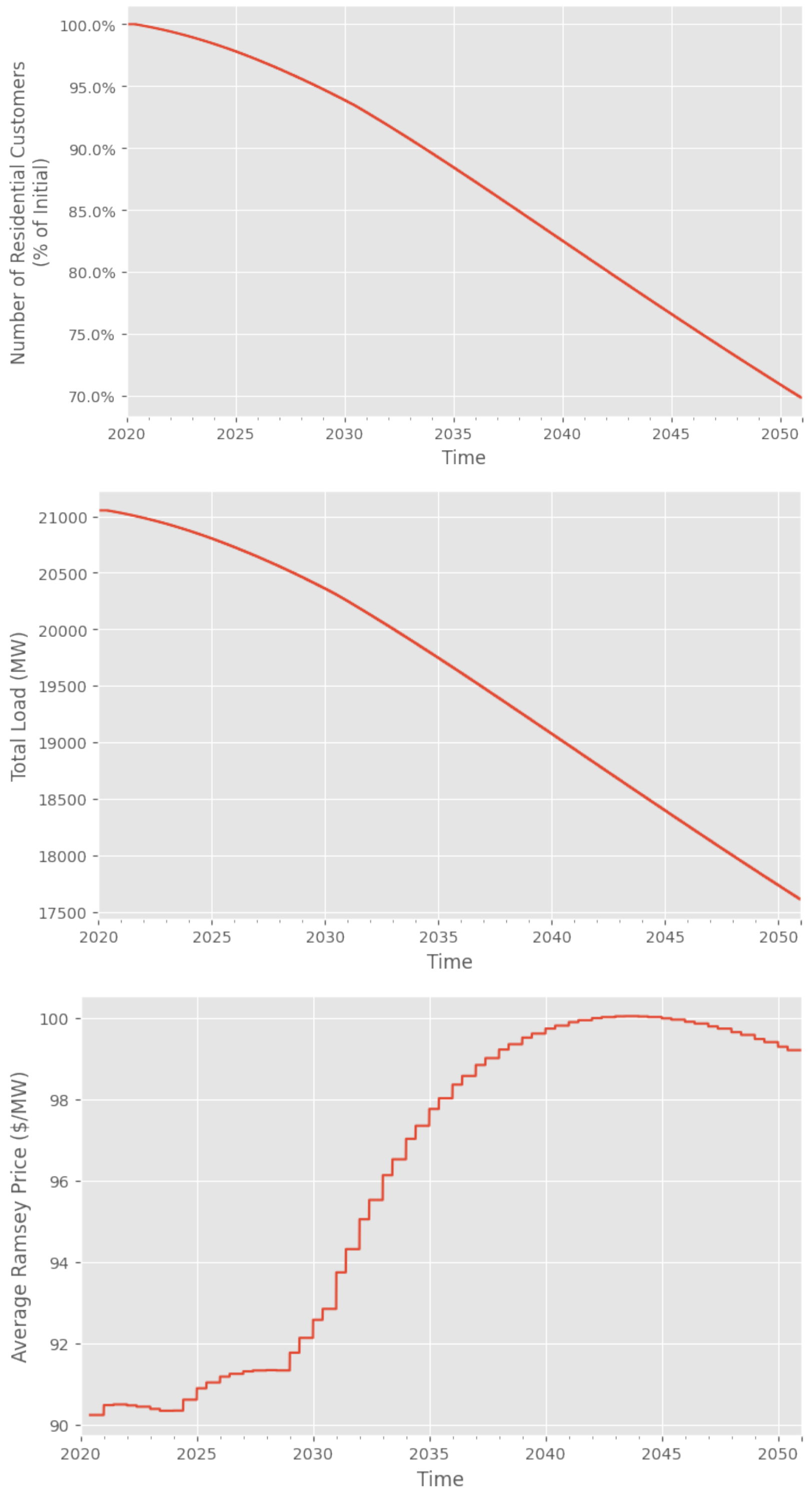

Appendix Figure 6: Benchmark simulation results for the TVA service area. Benchmark scenario includes a 26% federal rebate on the cost of solar panels, 4 sun-hours of solar irradiance, and planned coal-fired generator retirements.

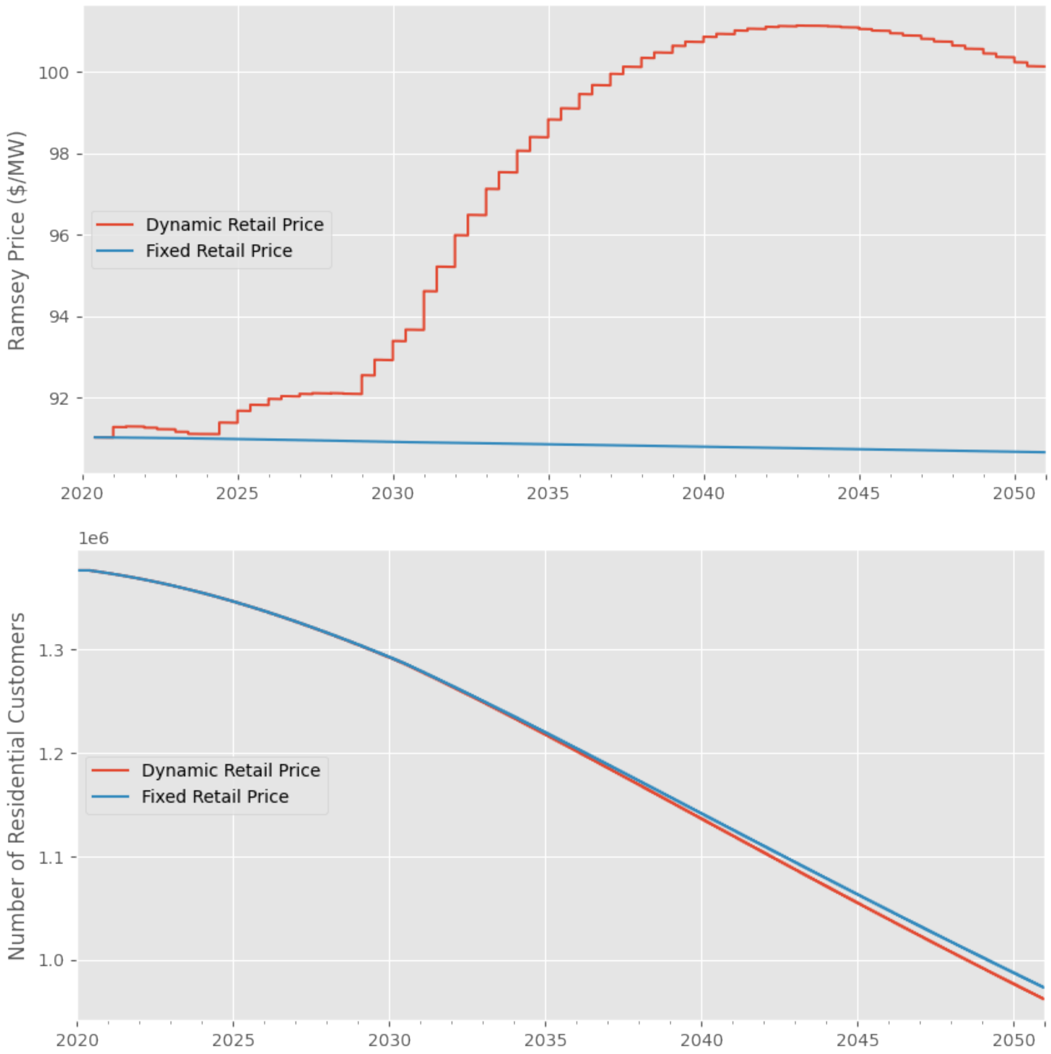

Appendix Figure 7: Comparing fixed price and dynamic price simulation results to identify the additional customers that defect from the grid due to the utility death spiral in the TVA service area

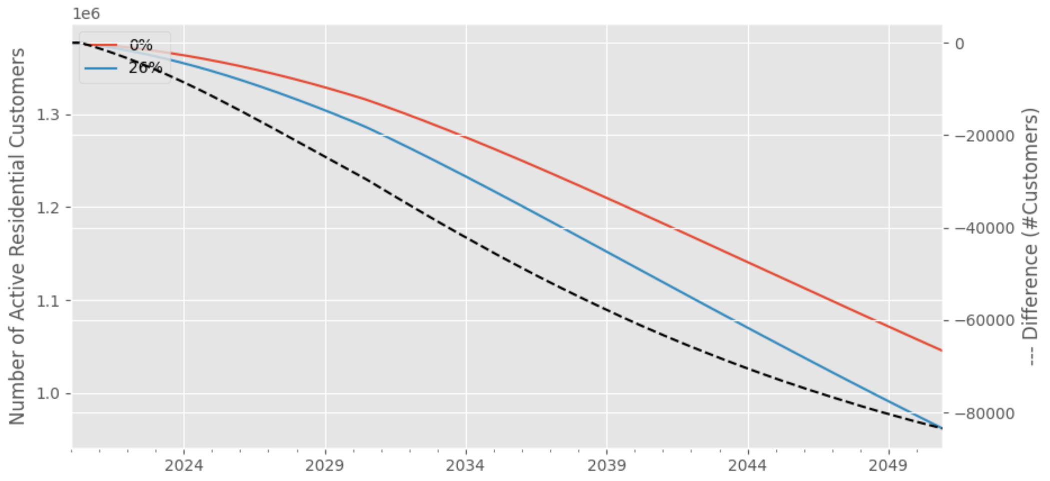

Appendix Figure 8: Comparing Rebates: 26% Rebates vs No Rebates

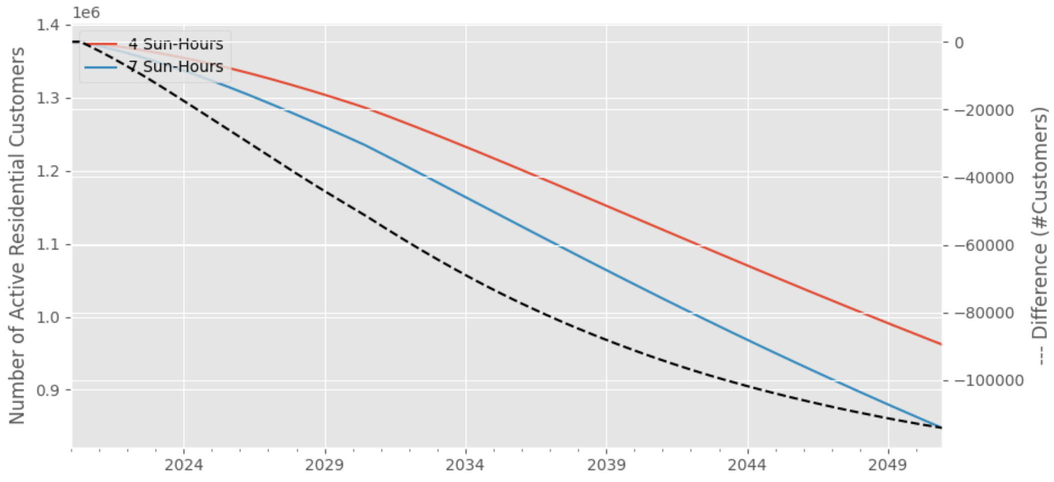

Appendix Figure 9: Comparing Sun Hours: 4 Sun-Hours vs. 7 Sun-Hours

6 References

Barbose, G., Darghouth, N., Elmallah, S., Forrester, S., Kristina S. H., K., Millstein, D., Rand, J., Cotton, W., Sherwood, S., and O'Shaughnessy, E. (2019). "Tracking the Sun: Pricing and Design Trends for Distributed Photovoltaic Systems in the United States - 2019 Edition." Lawrence Berkeley National Lab.(LBNL), Berkeley, CA (United States)

Barbose, G., Darghouth, N., O'Shaughnessy, E., and Forrester, S. (2023). "Tracking the Sun: Pricing and Design Trends for Distributed Photovoltaic Systems in the United States - 2023 Edition." Lawrence Berkeley National Lab.(LBNL), Berkeley, CA (United States)

BloombergNEF. (2019). "A Behind the Scenes Take on Lithium-ion Battery Prices." BloombergNEF.

Boiteux, M. (1956). Sur la gestion des monopoles publics astreints à l'équilibre budgétaire. Econometrica, Journal of the Econometric Society, 22-40.

Burke, Paul J., and Ashani Abayasekara. "The price elasticity of electricity demand in the United States: A three-dimensional analysis." The Energy Journal 39.2 (2018): 123-146.

Dong, C., and Sigrin, B. (2019). Using willingness to pay to forecast the adoption of solar photovoltaics: A "parameterization+ calibration" approach. Energy Policy, 129, 100-110.

Espey, James A., and Molly Espey. "Turning on the lights: A meta-analysis of residential electricity demand elasticities." Journal of Agricultural and Applied Economics 36.1 (2004): 65-81.

Feldman, D. J., and Schwabe, P. D. (2018). Terms, trends, and insights on PV project finance in the United States, 2018 (No. NREL/TP-6A20-72037). National Renewable Energy Lab.(NREL), Golden, CO (United States).

Goldfarb, D., and A. Idnani. "A Numerically Stable Dual Method for Solving Strictly Convex Quadratic Programs." Mathematical Programming 27, no. 1 (September 1, 1983): 1–33. https://doi.org/10.1007/BF02591962.

Haruna, H., Itoh, S., Horiba, T., Seki, E., and Kohno, K. (2011). Large-format lithium-ion batteries for electric power storage. Journal of Power Sources, 196(16), 7002-7005.

Ito, K. (2014). Do consumers respond to marginal or average price? Evidence from nonlinear electricity pricing. American Economic Review, 104(2), 537-563.

McFadden D. 1974. Conditional logit analysis of qualitative choice behavior. In Frontiers of Econometrics, ed. P Zarembka, pp. 105–42. New York: Academic

NREL PVWatts Calculator. 2021. url: https//web.archive.org/web/20210825182440/https://pvwatts.nrel.gov/pvwatts.p

(visited on 08/25/2021).

Omar, N., Firouz, Y., Gualous, H., Salminen, J., Kallio, T., Timmermans, J. M., ... Van Mierlo, J. (2015). Aging and degradation of lithium-ion batteries. In Rechargeable lithium batteries (pp. 263-279). Woodhead Publishing.

Petrovich, B., Carattini, S., and Wüstenhagen, R. (2021). The price of risk in residential solar investments. Ecological Economics, 180, 106856.

Ramsey, F. P. (1927). A Contribution to the Theory of Taxation. The Economic Journal, 37(145), 47-61.

Sun, J., and Tesfatsion, L., 2007. An Agent-Based Computational Laboratory for Wholesale Power Market Design. 2007 IEEE Power Engineering Society General Meeting, IEEE, Tampa, FL, USA, 1–6.

Sun, J., and Tesfatsion, L. (2010). DC optimal power flow formulation and solution using QuadProgJ.

Tervo, E., Agbim, K., DeAngelis, F., Hernandez, J., Kim, H. K., Odukomaiya, A. (2018). An economic analysis of residential photovoltaic systems with lithium ion battery storage in the United States. Renewable and Sustainable Energy Reviews, 94, 1057-1066.

Zhu, X., Li, L., Zhou, K., Zhang, X., and Yang, S. (2018). A meta-analysis on the price elasticity and income elasticity of residential electricity demand. Journal of Cleaner Production, 201, 169-177.
